# Analysis of the Expression and Prognostic Potential of a Novel Metabolic Regulator ANGPTL8/Betatrophin in Human Cancers

**DOI:** 10.3389/pore.2021.1609914

**Published:** 2021-09-27

**Authors:** Fangfang Xu, Dandan Tian, Xiaoyang Shi, Kai Sun, Yuqing Chen

**Affiliations:** ^1^ Clinical Medical Research Center, Henan Provincial People’s Hospital and Zhengzhou University People’s Hospital, Zheng Zhou, China; ^2^ Department of Hypertension, Henan Provincial People’s Hospital and Zhengzhou University People’s Hospital, Zheng Zhou, China; ^3^ Department of Endocrinology, Henan Provincial People’s Hospital and Zhengzhou University People’s Hospital, Zheng Zhou, China; ^4^ Department of Hematology, Henan Provincial People’s Hospital and Zhengzhou University People’s Hospital, Zheng Zhou, China

**Keywords:** inflammation, prognostic biomarker, ANGPTL8/betatrophin, differential expression analysis, lipid metabolism, glucose homeostasis, Akt signaling

## Abstract

The angiopoietin-like protein (ANGPTL) family members, except for the novel atypical member ANGPTL8/betatrophin, have been reported to participate in angiogenesis, inflammation and cancer. ANGPTL8/betatrophin is a metabolic regulator that is involved in lipid metabolism and glucose homeostasis. However, little is known about the expression and prognostic value of ANGPTL8/betatrophin in human cancers. In this study, we first conducted detailed analyses of ANGPTL8/betatrophin expression in cancer/normal samples *via* the Human Protein Atlas (HPA), Gene Expression Profiling Interactive Analysis (GEPIA), DriverDBv3, ENCORI and UALCAN databases. ANGPTL8/betatrophin showed high tissue specificity (enriched in the liver) and cell-type specificity (enriched in HepG2 and MCF7 cell lines). More than one databases demonstrated that the gene expression of ANGPTL8/betatrophin was significantly lower in cholangiocarcinoma (CHOL), breast invasive carcinoma (BRCA), lung adenocarcinoma (LUAD), lung squamous cell carcinoma (LUSC), uterine corpus endometrial carcinoma (UCEC), and significantly higher in kidney renal clear cell carcinoma (KIRC) compared with that in normal samples. However, the protein expression of ANGPTL8/betatrophin displayed opposite results in clear cell renal cell carcinoma (ccRCC)/KIRC. Based on the expression profiles, the prognostic value was evaluated with the GEPIA, DriverDBv3, Kaplan Meier plotter and ENCORI databases. Two or more databases demonstrated that ANGPTL8/betatrophin significantly affected the survival of KIRC, uterine corpus endometrial carcinoma (UCEC), pheochromocytoma and paraganglioma (PCPG) and sarcoma (SARC); patients with PCPG and SARC may benifit from high ANGPTL8/betatrophin expression while high ANGPTL8/betatrophin expression was associated with poor prognosis in KIRC and UCEC. Functional analyses with the GeneMANIA, Metascape and STRING databases suggested that ANGPTL8/betatrophin was mainly involved in lipid homeostasis, especially triglyceride and cholesterol metabolism; glucose homeostasis, especially insulin resistance; AMPK signaling pathway; PI3K/Akt signaling pathway; PPAR signaling pathway; mTOR signaling pathway; HIF-1 signaling pathway; autophagy; regulation of inflammatory response. ANGPTL8/betatrophin may be a promising prognostic biomarker and therapeutic target, thus providing evidence to support further exploration of its role in defined human cancers.

## Introduction

Betatrophin, also known as hepatocellular carcinoma-associated protein TD26, lipasin, refeeding induced fat and liver (RIFL), atypical angiopoietin-like protein 8 (ANGPTL8) or C19orf80 [1–3], is a newly identified secreted metabolic regulator predominantly expressed in the human liver. In the present study, it was referred to as ANGPTL8/betatrophin. Previous studies have shown that ANGPTL8/betatrophin is involved in the regulation of both lipid metabolism and glucose homeostasis [3–6]. It is well known that cancer cells change their metabolism to support rapid proliferation and expansion; hence, abnormal lipid and glucose metabolism has been found to be involved in a variety of cancers [7–11]. The angiopoietin-like protein (ANGPTL) family consists of eight members, ANGPTL1-8. ANGPTL2, 3, 4, 6, and 7 are known as proinflammatory factors that regulate cancer progression, while ANGPTL1 inhibits tumor angiogenesis and metastasis **[**12]. However, we do not know whether the atypical ANGPTL family member ANGPTL8/betatrophin also plays a role in human cancers.

Previous studies have reported that ANGPTL8/betatrophin improves insulin sensitivity *via* the insulin-mediated AKT phosphorylation, the activation of Akt-GSK3β or Akt-FoxO1 pathway **[**13, 14]. The Akt is frequently deregulated in many cancers and associated with the proliferation and survival of cancer cells [15–17]. It suggested a role of ANGPTL8/betatrophin in human cancer. Recent studies found that the serum ANGPTL8/betatrophin concentrations were significantly higher in hepatocellular carcinoma patients than in healthy controls **[**18]. ANGPTL8/betatrophin may act as a moderate suppressor of hepatocellular carcinoma **[**19]. However, the circulating levels of ANGPTL8/betatrophin were negatively correlated with renal function **[**20]. Furthermore, the 5-year survival rate of kidney renal clear cell carcinoma (KIRC) patients with high ANGPTL8/betatrophin expression is decreased significantly compared with that of patients with low ANGPTL8/betatrophin expression (51 vs. 78%, *p* = 7.0e-7), as estimated by the Pathology Atlas in the Human Protein Atlas (HPA) database. Based on its association with a poor outcome, ANGPTL8/betatrophin may serve as a novel unfavorable prognostic marker in renal cancer. These results indicate the prognostic potential of ANGPTL8/betatrophin in human cancers, which has yet to be defined.

In the present study, we comprehensively analyzed ANGPTL8/betatrophin expression and the correlation between the expression level of ANGPTL8/betatrophin and the survival of cancer patients to explore its prognostic value *via* online databases or web tools, including the HPA, GEPIA, DriverDBv3, ENCORI, UALCAN, and Kaplan Meier plotter. The GeneMANIA, Metascape and STRING databases were used to explore the functional network and the potential mechanism of ANGPTL8/betatrophin in cancers. Unexpectedly, through these analyses, we found a correlation between ANGPTL8/betatrophin expression and prognosis not only in KIRC but also in PCPG, SARC and UCEC. These results suggests that ANGPTL8/betatrophin may be a potential prognostic biomarker and promising therapeutic target in certain cancer types.

## Materials and Methods

### Expression Analysis of ANGPTL8/Betatrophin in the Human Protein Atlas Database

The Human Protein Atlas (HPA) database aims to map human proteins in organs, tissues and cells *via* various omics technologies and all the data is open access (http://www.proteinatlas.org). The tissue RNA expression levels from 55 tissue types and 6 blood cell types in the Tissue Atlas, the cell line RNA expression levels from 64 cell lines in the Cell Atlas, and cancer tissue expression levels across 17 main cancer types of ANGPTL8/betatrophin, as well as correlation analysis of ANGPTL8/betatrophin expression and patient survival the Pathology Atlas were analyzed.

### Expression and Survival Analyses Using the Gene Expression Profiling Interactive Analysis Database

The Gene Expression Profiling Interactive Analysis (GEPIA) database was used to analyze the RNA sequencing expression data of both tumor and normal samples from The Cancer Genome Atlas (TCGA) and Genotype-Tissue Expression (GTEx) projects **[**21]. GEPIA was used to perform tumor/normal differential expression analysis, expression profiling according to cancer types or pathological stages, and survival analysis of ANGPTL8/betatrophin in 33 cancer types. The hazard ratio (HR) and longrank *p* value were included in the plot. A longrank *p* value less than 0.05 was considered to indicate a statistically significant difference.

### Expression and Survival Analyses in the DriverDBv3 Database

The DriverDBv3 database provides RNA expression, somatic mutation, methylation and survival data of a selected gene across multiple cancer types **[**22]. The “Cancer,” “Gene,” and “Customized-Analysis” functions in DriverDBv3 database help to visualize the relationships between ANGPTL8/betatrophin and cancers.

### Expression and Survival Analyses in the Encyclopedia of RNA Interactomes Database

The Encyclopedia of RNA Interactomes (ENCORI) database is now not only focus on miRNA-target interactions but also provides gene expression data of 32 cancer types. ENCORI Pan-Cancer Analysis Platform allows researchers to perform gene differential expression analysis and gene survival analysis across 32 cancer types integrated from TCGA project.

### Expression and Survival Analyses Using the UALCAN Database

The UALCAN database was also used to explore the gene and protein expression and survival information of ANGPTL8/betatrophin in different cancer types. UALCAN is a comprehensive web resource designed to identify biomarkers and provides gene expression and survival information based on gene expression **[**23]. Besides, UALCAN now provides a protein expression analysis across 7 cancers (with tumor and normal samples) using data from the Clinical Proteomic Tumor Analysis Consortium (CPTAC) dataset **[**24].

### Survival Analysis Using the Kaplan Meier Plotter Database

The Kaplan Meier (KM) plotter database was used to assess the effect of ANGPTL8/betatrophin on survival in 21 cancer types. Gene expression data and survival information in KM plotter are downloaded from TCGA, Gene Expression Omnibus (GEO), and European Genome-phenome Archive (EGA) datasets **[**25]. The logrank *p* value less than 0.05 was considered statistically significant.

### Protein-Protein Interaction Network Construction and Functional Enrichment Analyses

The protein-protein interaction (PPI) network and function of ANGPTL8/betatrophin were predicted using the GeneMANIA, Metascape, Search Tool for the Retrieval of Interacting Genes/Proteins (STRING) databases.

GeneMANIA is an online tool that can help to predict gene function. GeneMANIA finds other genes that are related to the input gene using a very large set of functional association data, including data on protein and gene interactions, pathways, coexpression, colocalization and gene enrichment **[**26]. These genes interacting with ANGPTL8/betatrophin in the GeneMANIA network were all imported into Metascape for gene list annotation and analysis **[**27].

The STRING database provides known and predicted associations between proteins, including direct (physical) interactions and indirect (functional) associations; STRING collects and scores evidence from the scientific literature, databases of interaction experiments and annotated pathways or complexes, computational interaction predictions, and systematic transfers of interaction evidence between organisms **[**28].

## Results

### The Expression Profiles of ANGPTL8/Betatrophin in Human Cancers

To assess the differences in ANGPTL8/betatrophin expression in/between tumor and normal samples, its expression profiles were determined using the HPA, GEPIA, DriverDBv3, ENCORI and UALCAN databases. Gene symbols for ANGPTL8/betatrophin identified in each database were shown in Table 1 and tumor abbreviations were listed in Supplementary Table S1. The other options were all system default.

**TABLE 1:** Gene symbols for ANGPTL8/betatrophin identified in each database.

database	Gene symbol
HPA	ANGPTL8
GEPIA	C19orf80
DriverDBv3	ANGPTL8
ENCORI	ANGPTL8
KM plotter	ANGPTL8
UALCAN	ANGPTL8
STRING	C19orf80

In the HPA database, the expression of ANGPTL8/betatrophin was enriched in the liver across 55 tissue types and 6 blood cell types (Supplementary Figure S1). In addition, it was also slightly expressed in the adipose tissue, colon, breast, salivary gland, lymph node, urinary bladder and kidney (Supplementary Figure S1). The expression of ANGPTL8/betatrophin was enriched in HepG2 and MCF7 cells across the 69 cell lines assessed (Supplementary Figure S2 and Supplementary Figure S3). ANGPTL8/betatrophin was detected in and mainly localized to the Golgi apparatus; it was also expressed in the nucleoplasm (Supplementary Figure S3) and was predicted to be secreted partially based on the N-terminal signal sequence (signal peptide). The results of the Pathology Atlas also showed that ANGPTL8/betatrophin was enriched in liver cancer across 17 cancer types (Supplementary Figure S4).

**FIGURE 1:**
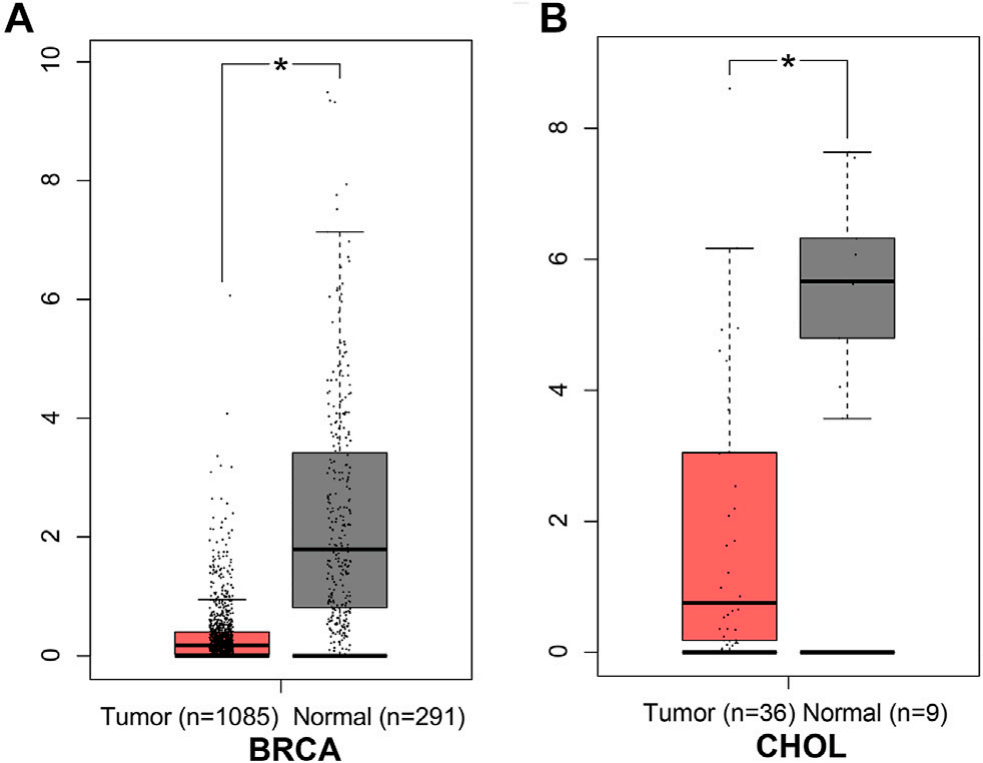
ANGPTL8/betatrophin expression between tumor and normal samples determined by the GEPIA database. GEPIA, Gene Expression Profiling Interactive Analysis; BRCA, Breast invasive carcinoma; CHOL, Cholangiocarcinoma; *p < 0.05.

In the GEPIA database, different expression levels of ANGPTL8/betatrophin were observed across 33 cancer types (Supplementary Figure S5). ANGPTL8/betatrophin was enriched in liver hepatocellular carcinoma (LIHC) as well as normal controls (Supplementary Figure S5), and was significantly lower in breast invasive carcinoma (BRCA) and cholangiocarcinoma (CHOL) than in normal samples (Figure 1).

**FIGURE 2:**
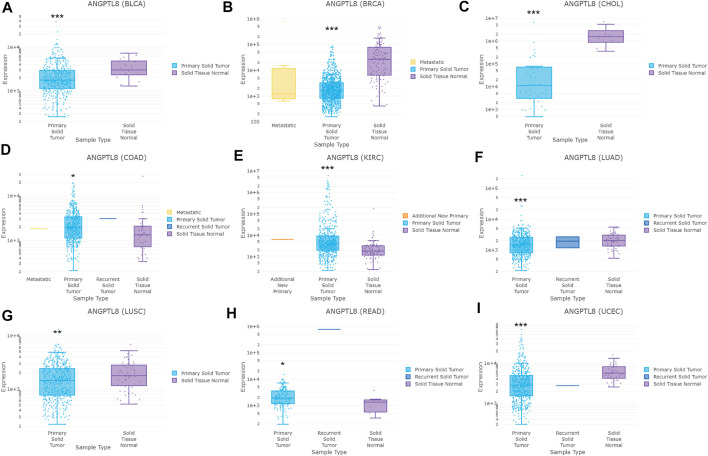
ANGPTL8/betatrophin expression between tumor and normal samples determined by the DriverDBv3 database. DriverDBv3, a database for human cancer driver gene research; BLCA, Bladder urothelial carcinoma; BRCA, Breast invasive carcinoma; CHOL, Cholangiocarcinoma; COAD, Colon adenocarcinoma; KIRC, Kidney renal clear cell carcinoma; LUAD, Lung adenocarcinoma; LUSC, Lung squamous cell carcinoma; READ, Rectum adenocarcinoma; UCEC, Uterine corpus endometrial carcinoma; *p < 0.05, **p < 0.01, ***p < 0.001 vs. normal.

In the DriverDBv3 database, different expression levels of ANGPTL8/betatrophin was found across 33 cancer types (Supplementary Figure S6). ANGPTL8/betatrophin was enriched in LIHC as well as normal tissues (Supplementary Figure S6), significantly lower in BRCA, CHOL, bladder urothelial carcinoma (BLCA), lung adenocarcinoma (LUAD), lung squamous cell carcinoma (LUSC), uterine corpus endometrial carcinoma (UCEC), and significantly higher in colon adenocarcinoma (COAD), kidney renal clear cell carcinoma (KIRC), rectum adenocarcinoma (READ) than in normal tissues (Figure 2).

**FIGURE 3:**
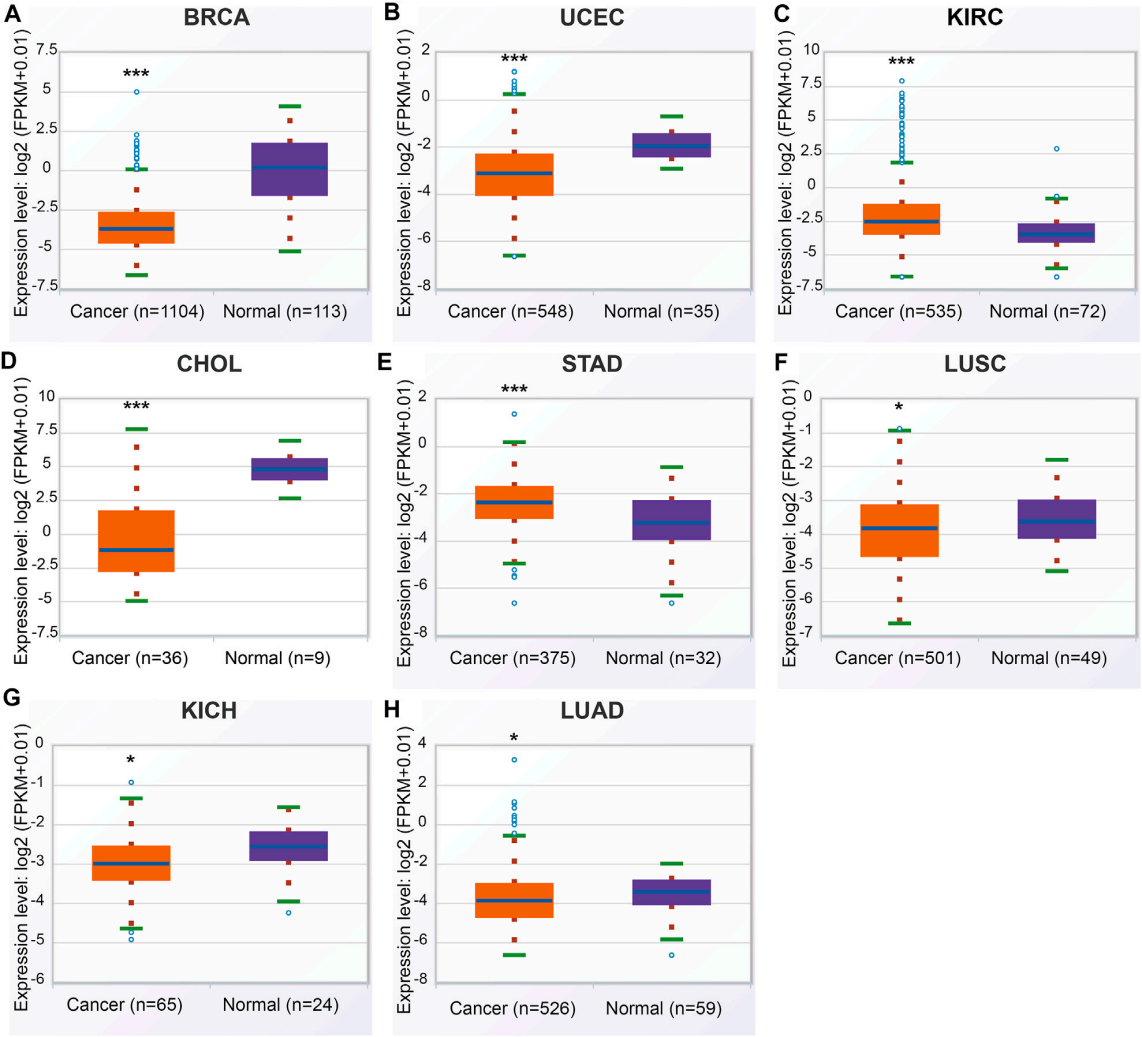
ANGPTL8/betatrophin expression between tumor and normal samples determined by the ENCORI database. ENCORI, The Encyclopedia of RNA Interactomes; BRCA, Breast invasive carcinoma; UCEC, Uterine corpus endometrial carcinoma; KIRC, Kidney renal clear cell carcinoma; CHOL, Cholangiocarcinoma; STAD, Stomach adenocarcinoma; LUSC, Lung squamous cell carcinoma; KICH, Kidney chromophobe; LUAD, Lung adenocarcinoma; *p < 0.05, ***p < 0.001 vs. normal.

In the ENCORI database, ANGPTL8/betatrophin was significantly lower in BRCA, CHOL, LUAD, LUSC, UCEC, kidney chromophobe (KICH), and significantly higher in KIRC, stomach adenocarcinoma (STAD) than in normal tissues across 32 cancer types (Figure 3).

**FIGURE 4:**
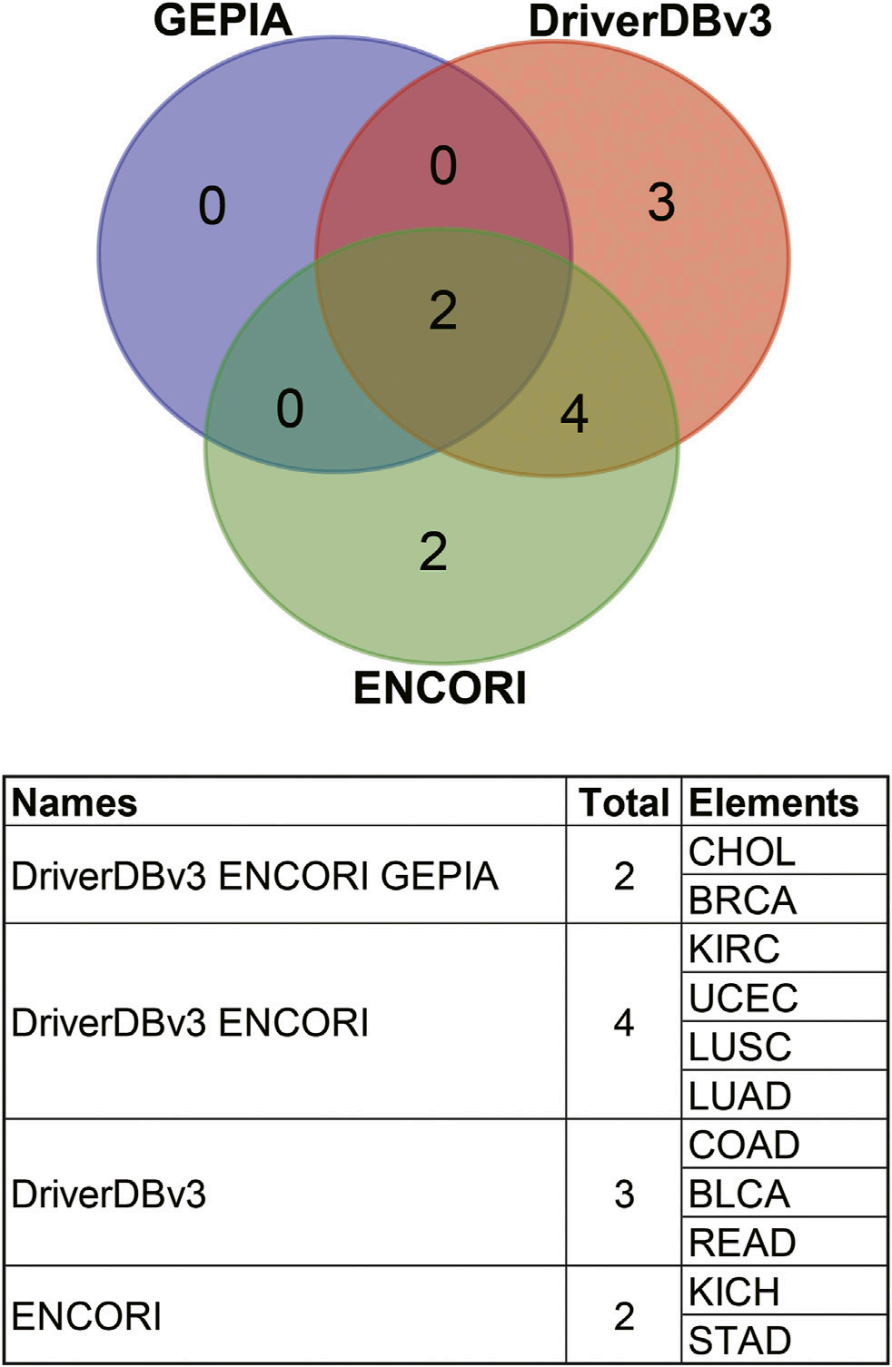
Comparisons of ANGPTL8/betatrophin expression profile resulted from different databases through a Venn diagram. GEPIA, Gene Expression Profiling Interactive Analysis; DriverDBv3: A database for human cancer driver gene research; ENCORI, The Encyclopedia of RNA Interactomes; CHOL, Cholangiocarcinoma; BRCA, Breast invasive carcinoma; KIRC, Kidney renal clear cell carcinoma; UCEC, Uterine corpus endometrial carcinoma; LUSC, Lung squamous cell carcinoma; LUAD, Lung adenocarcinoma; COAD, Colon adenocarcinoma; BLCA, Bladder urothelial carcinoma; READ, Rectum adenocarcinoma; KICH, Kidney chromophobe; STAD, Stomach adenocarcinoma.

Comparisons of the different expression profiles between the above databases were performed using a Venn diagram. Two or more databases demonstrated that ANGPTL8/betatrophin was significantly lower in BRCA, CHOL (GEPIA, DriverDBv3, and ENCORI), in LUAD, LUSC, and UCEC (DriverDBv3, ENCORI), and significantly higher in KIRC (DriverDBv3, ENCORI) than in normal tissues (Figure 4).

With regard to the protein expression profile of ANGPTL8/betatrophin in the UALCAN-CPTAC dataset, no expression information was available across 7 cancers except for clear cell renal cell carcinoma (ccRCC)/KIRC. Significant differences in protein expression levels of ANGPTL8/betatrophin were found in ccRCC/KIRC between tumor and normal samples (Supplementary Figure S7). Surprisingly, although the gene expression of ANGPTL8/betatrophin was obviously increased in ccRCC/KIRC (Figure 2E and Figure 3C), the protein expression of ANGPTL8/betatrophin was significantly lower than that in the normal samples (Supplementary Figure S7A). In addition, compared to that in the normal samples, ANGPTL8/betatrophin protein expression was significantly lower in ccRCC/KIRC samples from patients in cancer stage 1 and stage 3 (Supplementary Figure S7B), from Caucasian patients (Supplementary Figure S7C), from both males and females (Supplementary Figure S7D), from patients in the 21–40, 41–60 and 61–80 years old age groups (Supplementary Figure S7E), from patients in the extreme weight, obese and extreme obese categories (Supplementary Figure S7F), and from patients with tumor grade 2, grade 3 and grade 4 (Supplementary Figure S7G).

In terms of patient weight, as shown in Supplementary Figure S7F, there was also a significant difference in the expression of ANGPTL8/betatrophin between patients of normal weight and those in the extreme weight category, between patients of normal weight and those in the obese category, and between patients in the extreme weight and extreme obese categories. In terms of tumor grade, there was also an obvious difference in the expression of ANGPTL8/betatrophin between tumor grade 2 and grade 4 and between rumor grade 3 and grade 4 (Supplementary Figure S7G). The apparently contradictory results in gene and protein expression of ANGPTL8/betatrophin in ccRCC/KIRC need to be validated.

### Prognostic Potential of ANGPTL8/Betatrophin in Different Human Cancers

To investigate whether the expression level of ANGPTL8/betatrophin is correlated with the prognosis in cancer patients, its prognostic value was analyzed using the GEPIA, DriverDBv3, KM plotter and ENCORI databases.

**FIGURE 5:**
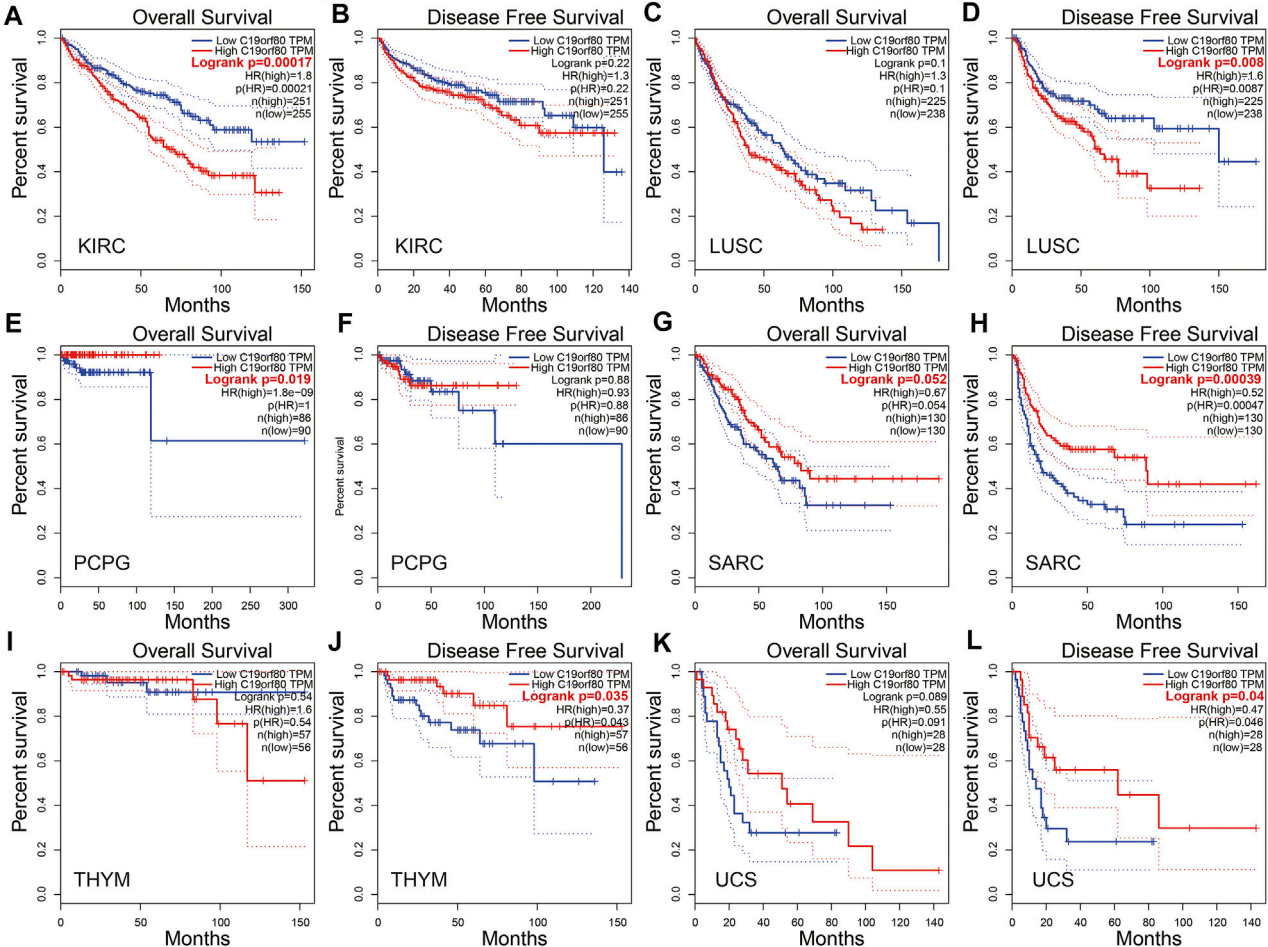
Survival analysis of ANGPTL8/betatrophin in cancer patients from the GEPIA database. Both the OS and DFS survival curves of **(A,B)** KIRC, **(C,D)** LUSC, **(E,F)** PCPG, **(G,H)** SARC, **(I,J)** THYM, **(K,L)** UCS were analyzed. GEPIA, Gene Expression Profiling Interactive Analysis; OS, overall survival; DFS, disease-free survival; KIRC, kidney renal clear cell carcinoma; LUSC, Lung squamous cell carcinoma; PCPG, pheochromocytoma and paraganglioma; SARC, Sarcoma; THYM, Thymoma; UCS, Uterine carcinosarcoma.

In the GEPIA database, ANGPTL8/betatrophin expression was significantly associated with overall survival (OS) and/or disease-free survival (DFS) in KIRC, LUSC, PCPG, SARC, THYM and UCS (Figure 5). The results revealed that high ANGPTL8/betatrophin expression was associated with poor prognosis in terms of OS in KIRC [OS logrank *p* = 0.00017, hazard ratio (HR) = 1.8; Figures 5A,B], and in terms of DFS in LUSC (DFS logrank *p* = 0.008, HR = 1.6; Figures 5C,D); however, high ANGPTL8/betatrophin expression was associated with favorable prognosis in terms of OS in PCPG (OS logrank *p* = 0.019, HR = 1.8e-09; Figures 4E,F), in terms of DFS in SARC (DFS logrank *p* = 0.00039, HR = 0.52; Figures 5G,H), THYM (DFS logrank *p* = 0.035, HR = 0.37; Figures 5I,J), and UCS (DFS logrank *p* = 0.04, HR = 0.47; Figures 5K,L).

**FIGURE 6:**
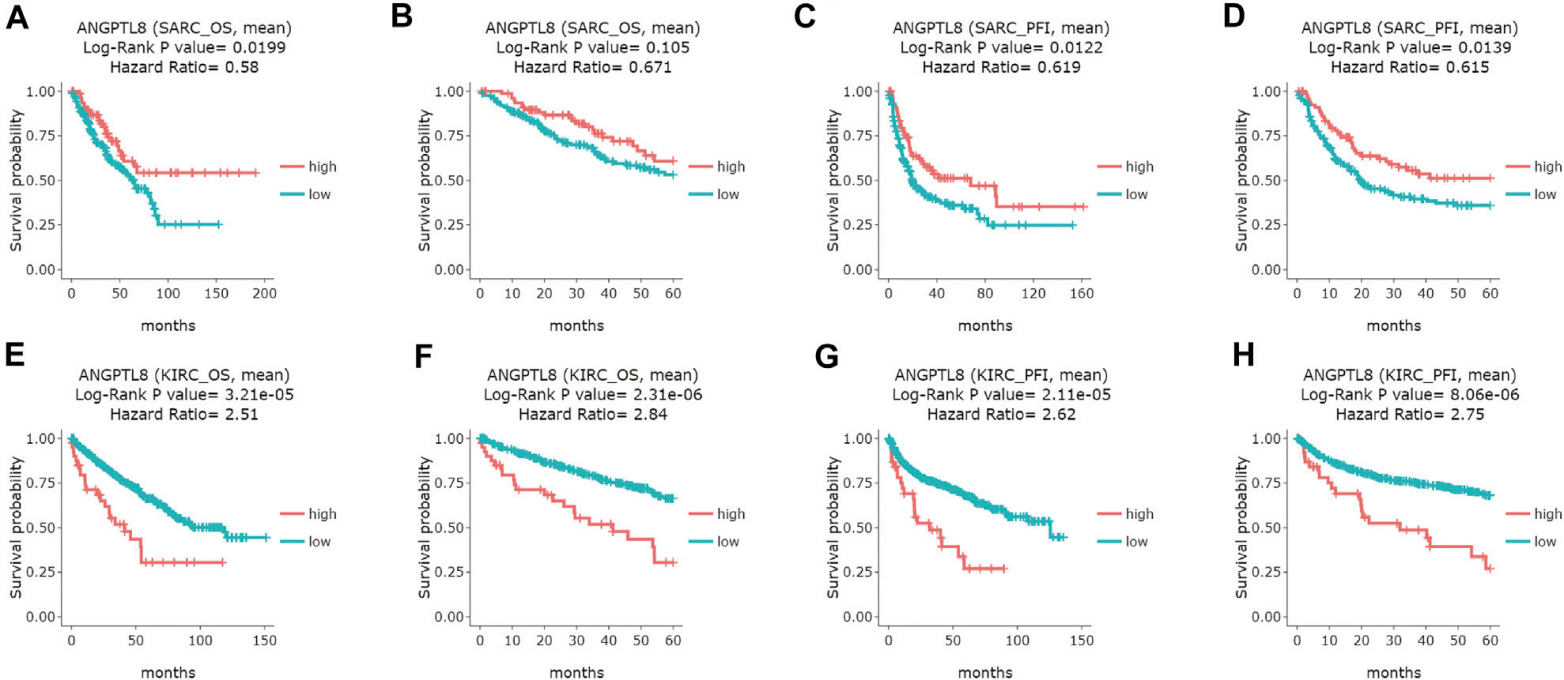
Survival analysis of ANGPTL8/betatrophin in cancer patients from the DriverDBv3 database. Overall survival and 5-years survival of both the OS and PFI survival curves of **(A–D)** SARC, **(E–H)** KIRC were analyzed. DriverDBv3, a database for human cancer driver gene research; OS, overall survival; PFI, progression-free interval; SARC, Sarcoma; KIRC, kidney renal clear cell carcinoma.

In the DriverDBv3 database, ANGPTL8/betatrophin expression was significantly associated with OS and/or progression-free interval (PFI) in SARC, KIRC, UVM, COAD and UCEC (Figure 6 and Supplementary Figure S8). High expression of ANGPTL8/betatrophin was associated with favorable prognosis in terms of OS (OS logrank *p* = 0.0199, HR = 0.58; Figure 6A) and PFI (OS logrank *p* = 0.0122, HR = 0.619; 5-years survival logrank *p* = 0.0139, HR = 0.615; Figures 6C,D) in SARC, and in terms of PFI in uveal melanoma [(UVM), OS logrank *p* = 0.0207, HR = 0.388; 5-years survival logrank *p* = 0.0207, HR = 0.388; Supplementary Figures S8A,B]; however, ANGPTL8/betatrophin high expression was associated with poor prognosis in terms of OS (OS logrank *p* = 3.21e-05, HR = 2.51; 5-years survival logrank *p* = 2.31e-06, HR = 2.84; Figures 6E,F) and PFI (OS logrank *p* = 2.11e-05, HR = 2.62; 5-years survival logrank *p* = 8.06e-06, HR = 2.75; Figures 6G,H) in KIRC, in terms of PFI in COAD (OS logrank *p* = 0.0227, HR = 1.53; 5-years survival logrank *p* = 0.028, HR = 1.52; Supplementary Figures S8C,D) and in UCEC (OS logrank *p* = 0.0494, HR = 1.44; 5-years survival logrank *p* = 0.0287, HR = 1.51; Supplementary Figures S8E,F).

**FIGURE 7:**
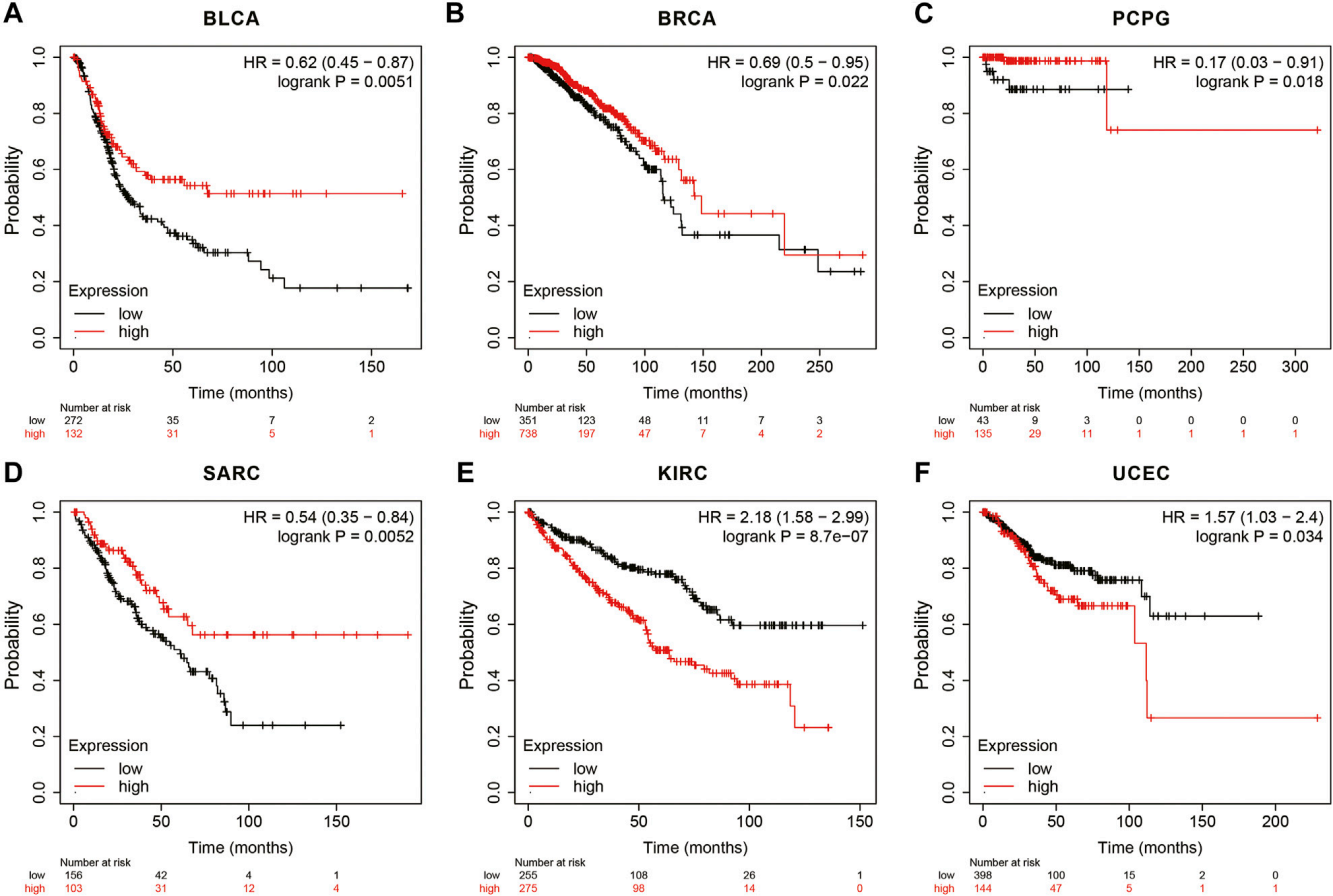
Survival analysis of ANGPTL8/betatrophin in cancer patients from the Kaplan Meier plotter database. OS survival curves of **(A)** BLCA, **(B)** BRCA, **(C)** PCPG, **(D)** SARC, **(E)** KIRC, **(F)** UCEC were analyzed. BLCA, Bladder urothelial carcinoma; BRCA, Breast invasive carcinoma; PCPG, Pheochromocytoma and paragangliomas; SARC, Sarcoma; KIRC, Kidney renal clear cell carcinoma; UCEC, Uterine corpus endometrial carcinoma.

In the Kaplan Meier plotter database, the results showed that high ANGPTL8/betatrophin expression was associated with favorable prognosis in terms of OS in BLCA (OS logrank *p* = 0.0051, HR = 0.62; Figure 7A), BRCA (OS logrank *p* = 0.0215, HR = 0.69; Figure 7B), PCPG (OS logrank *p* = 0.0183, HR = 0.17; Figure 7C), SARC (OS logrank *p* = 0.0052, HR = 0.54; Figure 7D). But in KIRC (OS logrank *p* = 8.70e-07, HR = 2.18; Figure 7E) and UCEC (OS logrank *p* = 0.0341, HR = 1.57; Figure 7F), high ANGPTL8/betatrophin expression was associated with poor prognosis.

**FIGURE 8:**
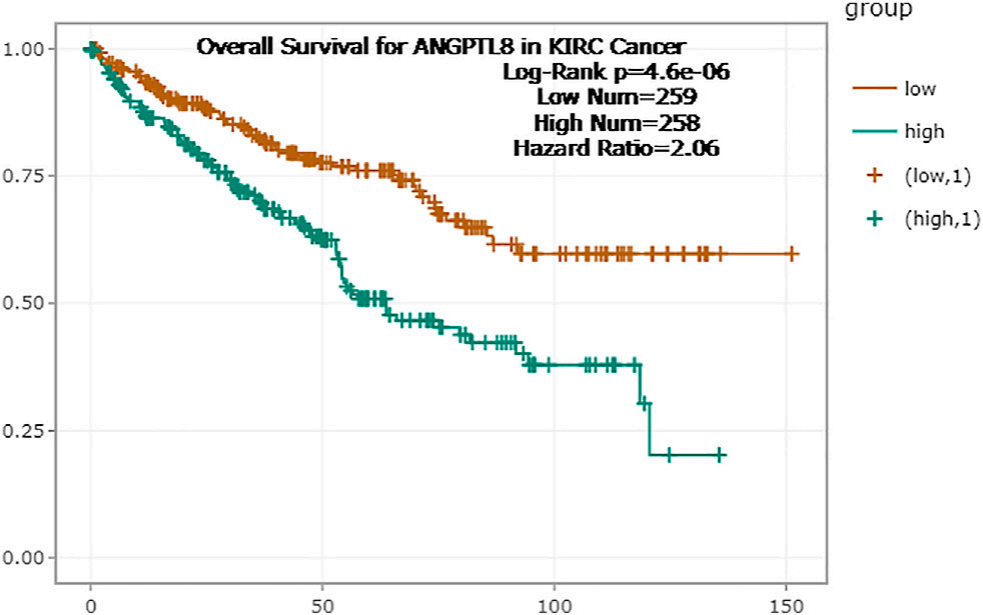
Survival analysis of ANGPTL8/betatrophin in cancer patients from the ENCORI database. ENCORI, The Encyclopedia of RNA Interactomes; KIRC, Kidney renal clear cell carcinoma.

In the ENCORI database, high expression of ANGPTL8/betatrophin was associated with poor prognosis in terms of OS (OS logrank *p* = 4.6e-6, HR = 2.06) in KIRC (Figure 8). Moreover, the Pathology Atlas in the HPA database also revealed that high expression of ANGPTL8/betatrophin was an unfavorable prognostic marker in renal cancer (https://www.proteinatlas.org/ENSG00000130173-ANGPTL8).

**FIGURE 9:**
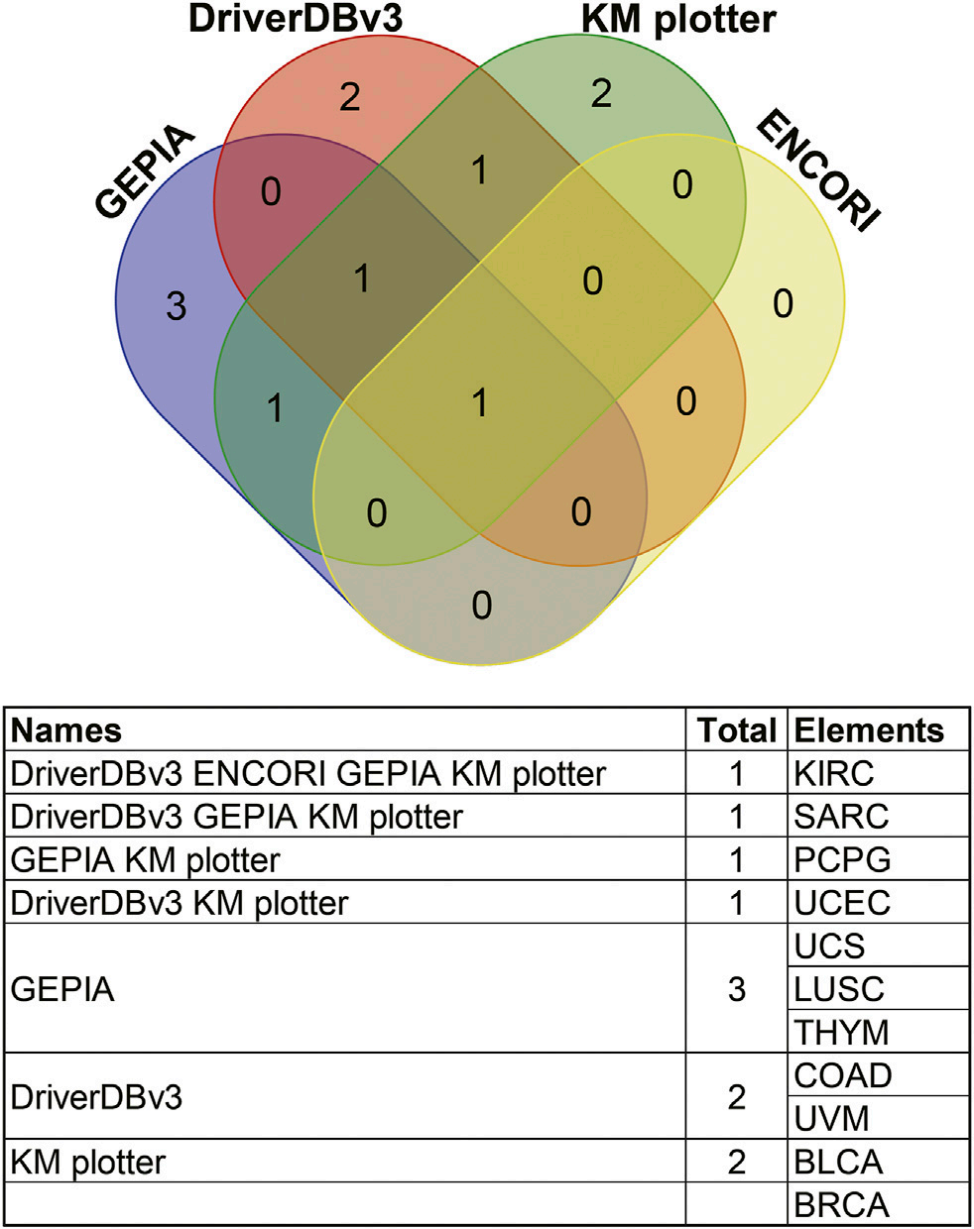
Comparisons of survival analyses of ANGPTL8/betatrophin between different databases through a Venn diagram. GEPIA, Gene Expression Profiling Interactive Analysis; DriverDBv3, a database for human cancer driver gene research; ENCORI, The Encyclopedia of RNA Interactomes; KM plotter, the Kaplan Meier plotter database; KIRC, Kidney renal clear cell carcinoma; SARC, Sarcoma; PCPG, Pheochromocytoma and paragangliomas; UCEC, Uterine corpus endometrial carcinoma; UCS, Uterine carcinosarcoma; LUSC, Lung squamous cell carcinoma; THYM, Thymoma; COAD, Colon adenocarcinoma; UVM, Uveal melanoma; BLCA, Bladder urothelial carcinoma; BRCA, Breast invasive carcinoma.

Survival results were compared between the above databases using a Venn diagram (Figure 9). Two or more databases demonstrated that high expression of ANGPTL8/betatrophin was associated with unfavorable prognosis in KIRC (GEPIA, DriverDBv3, KM plotter, ENCORI) and UCEC (DriverDBv3, KM plotter), while with favorable prognosis in SARC (GEPIA, DriverDBv3, KM plotter) and PCPG (GEPIA, KM plotter). Among the 4 cancers, KIRC, SARC, UCEC and PCPG mentioned above, there was a significant difference in ANGPTL8/betatrophin expression between normal and KIRC, between normal and UCEC tissue samples (Supplementary Figure S9). Thus, the metabolic regulator ANGPTL8/betatrophin may serve as a potential prognostic biomarker in those cancers.

### Protein-Protein Interaction Network and Functional Enrichment Analyses

**FIGURE 10:**
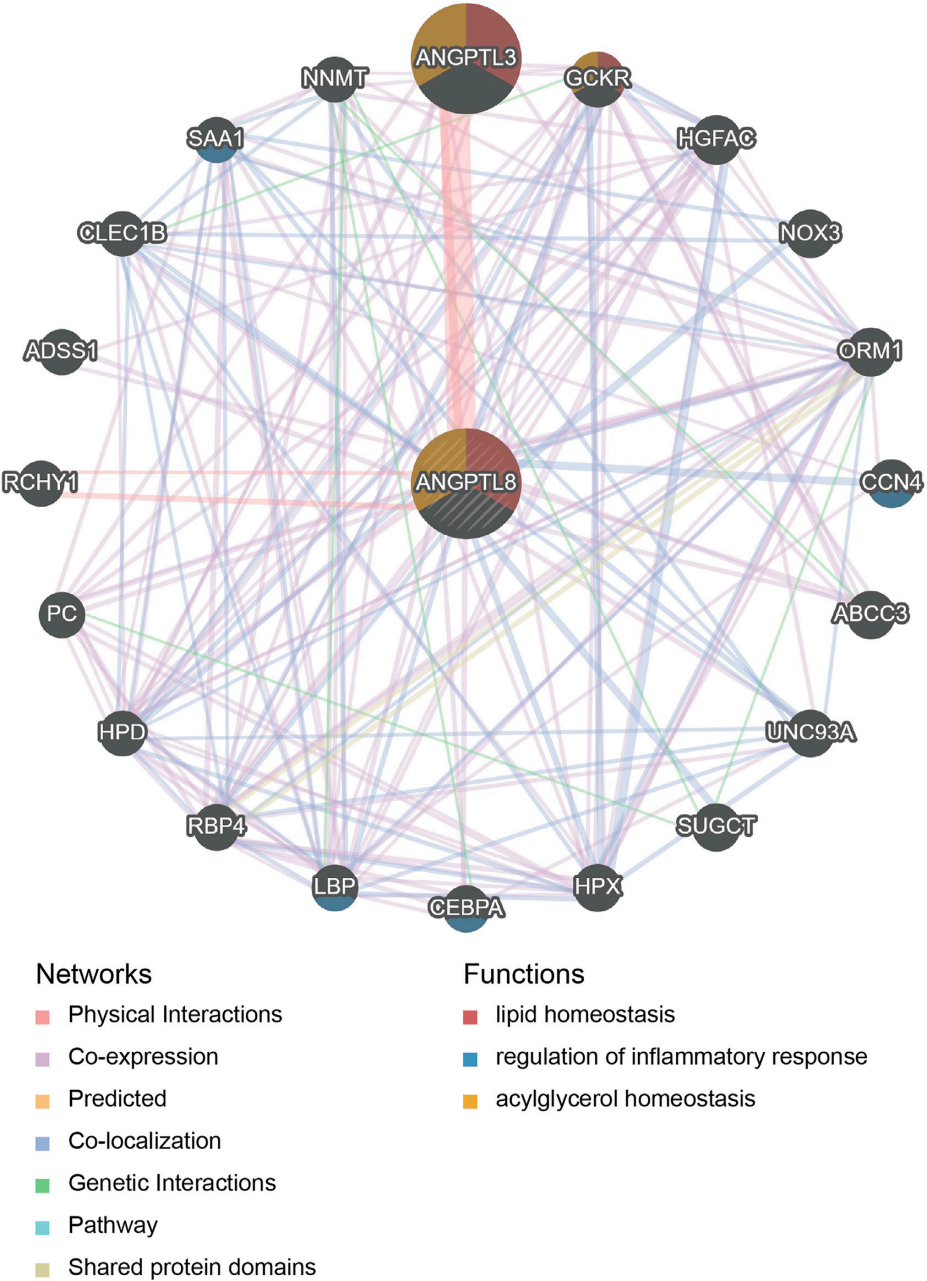
PPI network analysis of ANGPTL8/betatrophin *via* GeneMANIA. The database identified 20 genes closely associated with ANGPTL8/betatrophin. Colors of the networks indicate the data source from publicly available biological datasets: Physical Interaction, Co-expression, Predicted, Co-localization, Genetic interaction, Pathway, Shared protein domains. Colors of the network nodes indicate the functions of enrichment genes. PPI, protein-protein interaction.

**FIGURE 11:**
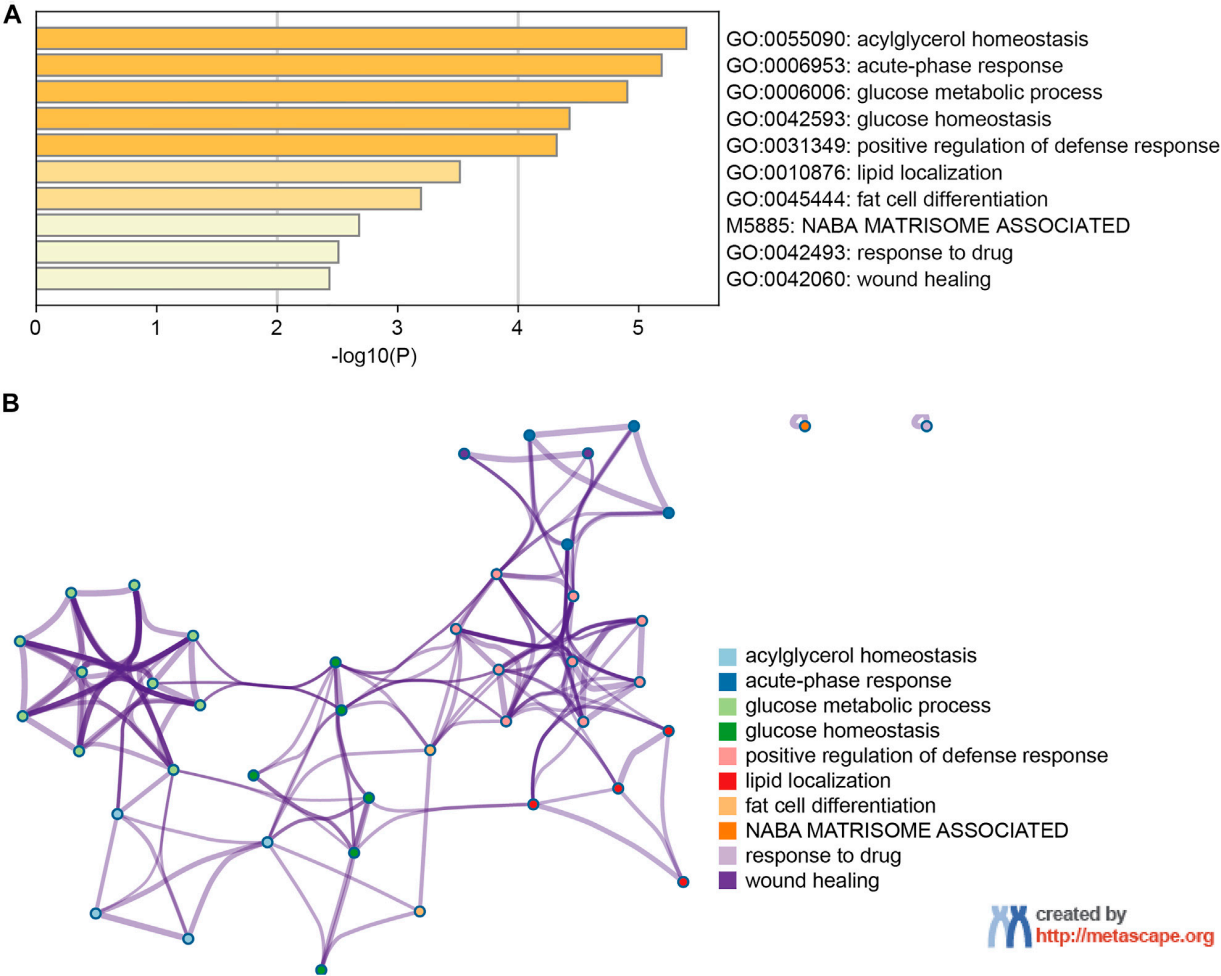
The enrichment analysis of ANGPTL8/betatrophin and the top 20 closely associated genes *via* Metascape. **(A)** Bar graph of top 10 enriched terms for ANGPTL8/betatrophin and the top 20 interacting genes, colored by *p* values. **(B)** Interactive network of 10 enriched terms colored by cluster ID where nodes that share the same cluster ID are typically close to each other.

PPI network and functional enrichment analyses were performed *via* GeneMANIA, Metascape and STRING databases. The PPI network analysis *via* GeneMANIA database revealed correlations between the top 20 genes and ANGPTL8/betatrophin (Table 2). The gene set enriched for ANGPTL8/betatrophin was related to lipid homeostasis, regulation of inflammatory response and acylglycerol homeostasis (Figure 10). The interactive genes of ANGPTL8/betatrophin were then input into the Metascape database for gene ontology (GO) and Kyoto Encyclopedia of Genes and Genomes (KEGG) pathway analyses, and the heatmap showed that GO biological processes including acylglycerol homeostasis, acute-phase response, glucose metabolic process, glucose homeostasis, positive regulation of defense response, lipid localization, fat cell differentiation were significantly regulated by these genes (Figure 11A). However, there was no output information about enriched KEGG pathways. These findings demonstrated that ANGPTL8/betatrophin plays an essential role in the response to stimulus, several metabolic processes, immune system process, and developmental process. To visualize the associations between the enriched terms, we constructed a network of enriched terms colored by cluster ID (Figure 11B).

**TABLE 2:** Genes interacting with ANGPTL8/betatrophin determined by the GeneMANIA database.

Symbol	Score	Functions
ANGPTL8	0.962150438	acylglycerol homeostasis, lipid homeostasis
ANGPTL3	0.001170777	acylglycerol homeostasis, lipid homeostasis
GCKR	0.000294697	acylglycerol homeostasis, lipid homeostasis
HGFAC	0.000206305	—
NOX3	0.000194931	—
ORM1	0.000194357	—
CCN4	0.000189535	regulation of inflammatory response
ABCC3	0.000185621	—
UNC93A	0.000179536	—
SUGCT	0.000176674	—
HPX	0.000171954	—
CEBPA	0.00016595	regulation of inflammatory response
LBP	0.000157241	regulation of inflammatory response
RBP4	0.000154189	—
HPD	0.00014915	—
PC	0.000148883	—
RCHY1	0.000147352	—
ADSS1	0.000146403	—
CLEC1B	0.000145764	—
SAA1	0.000142558	regulation of inflammatory response
NNMT	0.000141951	—

**FIGURE 12:**
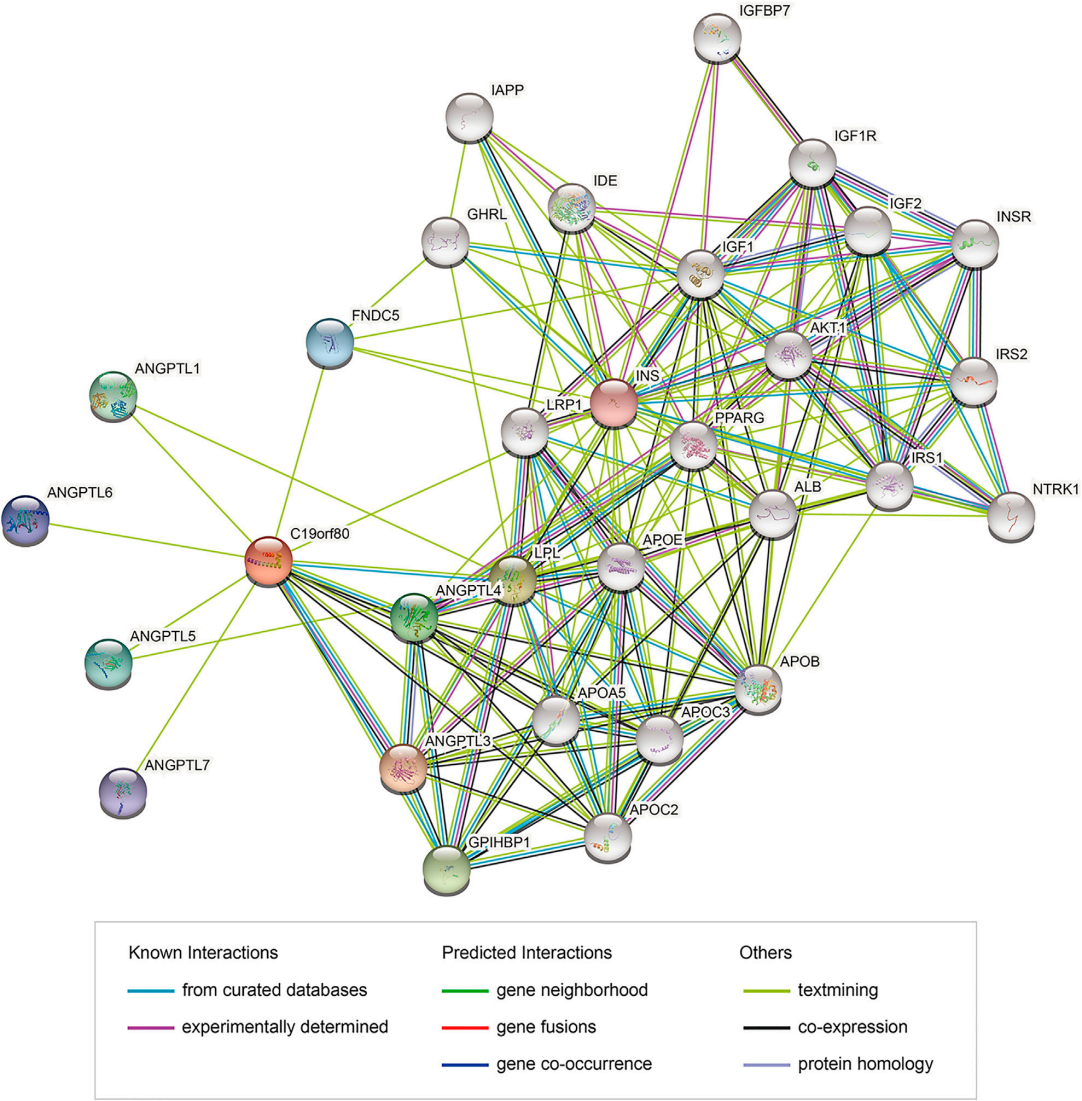
PPI network of genes associated with ANGPTL8/betatrophin using the STRING database. PPI, protein-protein interaction; STRING, Search Tool for the Retrieval of Interacting Genes; colored nodes: query proteins and first shell of interactors; white nodes: second shell of interactors; empty nodes: proteins of unknown 3D structure; filled nodes: some 3D structure is known or predicted.

PPI network analysis of ANGPTL8/betatrophin were also performed using the STRING database (Figure 12). GO biological processes, molecular functions and cellular components, and KEGG pathways were shown in Supplementary Data S1. The results showed that several biological processes including lipid homeostasis, glucose homeostasis, cellular response to endogenous stimulus, regulation of inflammatory response, and KEGG pathways including cholesterol metabolism, longevity regulating pathway, AMPK signaling pathway, PI3K/Akt signaling pathway, PPAR signaling pathway, mTOR signaling pathway, HIF-1 signaling pathway, insulin resistance, autophagy were significantly regulated by these genes. Although the list of ANGPTL8/betatrophin-interacting genes was different between the GeneMANIA and STRING databases, they were enriched in almost the same biological processes.

## Discussion

ANGPTL8/betatrophin seemes to be a pleiotropic hormone, as it was reported to regulate both lipid and glucose metabolism [2-6], and was recently reported to act as a moderate suppressor of human liver carcinoma cells [19]. ANGPTL8/betatrophin is not only enriched in the liver but is also reported to be expressed at significantly higher levels in hepatocellular carcinoma patients than in healthy controls [18]. The Pathology Atlas in the HPA database points out that ANGPTL8/betatrophin is an unfavorable prognostic marker in renal cancer. These results suggest that ANGPTL8/betatrophin may play a role in human cancers, which has not yet been well studied. In the present study, we first analyzed the expression profiles of ANGPTL8/betatrophin using the HPA, GEPIA, DriverDBv3, ENCORI and UALCAN databases, and then based on the results comprehensively performed survival analyses in different cancer types *via* various databases including GEPIA, DriverDBv3, Kaplan Meier plotter and ENCORI.

In normal tissues and cell lines, ANGPTL8/betatrophin showed high tissue specificity and cell-type specificity and was enriched in the liver and in the HepG2 and MCF7 cell lines. ANGPTL8/betatrophin exhibits liver-specific intragenic enhancer DNA hypomethylation, which explains why its expression is highest in the liver [29].

In tumor vs. normal samples, the gene expression of ANGPTL8/betatrophin showed great difference in several cancer types including BRCA, CHOL, LUAD, LUSC, UCEC, and KIRC. Correspondingly, ANGPTL8/betatrophin had a significant impact on the survival of UCEC and KIRC patients. Besides, ANGPTL8/betatrophin also showed great prognostic potential in SARC and PCPG even though there were no significant differences in the gene expression between tumor and normal samples. However, the association between ANGPTL8/betatrophin and the 6 cancers mentioned above has not yet been studied. Patients with SARC or PCPG may benifit from high ANGPTL8/betatrophin expression, in contrast, UCEC or KIRC patients may benifit from low expression of ANGPTL8/betatrophin.

KIRC, which has a poor prognosis, is the main histological subtype of renal cell carcinoma (RCC) [30]. High expression of ANGPTL8/betatrophin is an unfavourable prognostic marker in ccRCC/KIRC. The circulating levels of ANGPTL8/betatrophin were reported to be dependent on renal function [20]. It may be a novel endocrine regulator involved in diabetic nephropathy development and a biomarker of diabetic retinopathy [31, 32]. Although the gene expression of ANGPTL8/betatrophin was significantly higher in ccRCC/KIRC patients than in normal samples, surprisingly, the protein expression levels of ANGPTL8/betatrophin in ccRCC/KIRC of different sample types, individual cancer stages, patient races, patient gender, patient age groups, patient weight categories, tumor grades were significantly lower than that in normal samples in the UALCAN database CPTAC samples. The results are contradictory. Studies have shown that ANGPTL8/betatrophin, as a metabolic regulator, is significantly associated with age [33], body weight [34, 35], and gender [36]. Thus, it is necessary to experimentally validate both the gene and protein expression of ANGPTL8/betatrophin in ccRCC/KIRC patients as well as investigate the expression of ANGPTL8/betatrophin in the above-mentioned cancers grouped into those categories.

Gene functional analyses demonstrated that ANGPTL8/betatrophin was not only involved in metabolic pathways including lipid homeostasis, especially triglyceride and cholesterol metabolism, and glucose homeostasis, especially insulin resistance, but also in AMPK signaling pathway, PI3K/Akt signaling pathway, PPAR signaling pathway, mTOR signaling pathway, HIF-1 signaling pathway, autophagy, regulation of inflammatory response.

Metabolic reprogramming including glucose and fatty acid metabolism, and their crosstalk, is widely observed during cancer development to support cancer cell proliferation and invasiveness [37], which is regulated by HIF-1 and PI3K/Akt/mTOR pathway [38]. Target glucose metabolism may suppress cancer progression [38]. Studies have shown that ANGPTL8/betatrophin may regulate PI3K/Akt signaling pathway to alleviate insulin resistance [13, 14]. PI3K/Akt as well as AMPK signaling pathways have been reported to regulate ANGPTL8/betatrophin expression [39, 40].

Lipid metabolism is often dysregulated in cancer cells, and changes in lipid metabolism affect cellular processes such as proliferation, autophagy, and tumor development [41, 42]. The ANGPTL family members ANGPTL3, ANGPTL4 and ANGPTL8/betatrophin have emerged as key regulators of lipid metabolism that act by inhibiting the enzyme lipoprotein lipase (LPL) [43-45]. PPAR signaling is associated with cancer and exerts pleiotropic functions in cancer, as PPARs regulate lipid metabolism and insulin production, and PPAR genes are aberrantly expressed during cancer development [46, 47]. PPAR signaling may play a role in the regulation of ANGPTL8/betatrophin expression [48].

The ANGPTL family has also been reported to play important roles in angiogenesis, inflammation, and cancer and has recently been implicated in the regulation of stem cell activity [12, 49-52]. The circulating levels of ANGPTL8/betatrophin are increased under high levels of inflammation [53]. Inflammatory stimuli have been reported to induce the expression of ANGPTL8/betatrophin, which negatively regulates the activation of one of the most important central regulators of inflammation, nuclear factor-κB (NF-κB), to regulate inflammation [54]. Emerging evidence has shown the potential role of ANGPTL8/betatrophin in NF-κB mediated inflammation and autophagy [45]. The importance of NF-κB signaling in cancer development and the link between inflammation and cancer are well understood [55, 56], however, the role and mechanism of ANGPTL8/betatrophin in cancers need to be further studied.

This study has some limitations. First, databases or online tools enable large-scale genomics and functional analyses at low cost for researchers. However, different databases may yield different and even opposite results due to the difference in collected sample sizes and bioinformatics algorithms. Second, The current study only focused on the bioinformatics analysis of the prognostic role of ANGPTL8/betatrophin in cancers. Further dry lab analyses on the relationship between/among all ANGPTL family members and distinct human cancers and wet lab studies, including real-time quantitative polymerase chain reaction (RT-qPCR), western blot analysis and immunohistochemical examination, are required to validate these findings. Most importantly, the biological functions of ANGPTL8/betatrophin are not fully clarified, and further studies on its role in cancer progression, especially KIRC, UCEC, SARC and PCPG, should be performed in the near future.

## Conclusion

In the present work, we performed comprehensive analyses of the expression profiles and the correlation between the expression and prognostic value of the liver-derived metabolic regulator ANGPTL8/betatrophin in different cancers. Two or more databases demonstrated that the expression of ANGPTL8/betatrophin was significantly different in BRCA, CHOL, LUAD, LUSC, UCEC and KIRC, compared to that in normal samples; ANGPTL8/betatrophin greatly affected the survival of KIRC, UCEC, SARC and PCPG. ANGPTL8/betatrophin may be a potential prognostic biomarker in these cancers, which deserves further investigation. This will promote the clinical utility of ANGPTL8/betatrophin serving as a prognostic indicator or therapeutic target in certain cancers.

## Data Availability

The original contributions presented in the study are included in the article/Supplementary Material, further inquiries can be directed to the corresponding authors.

## Supplementary Material

Supplementary Figure S1 Tissue RNA expression of ANGPTL8/betatrophin in the HPA database 501 tissue atlas. HPA, Human Protein Atlas; NX, consensus normalized expression.

Supplementary Figure S2 Cell line RNA expression of ANGPTL8/betatrophin in the HPA database cell atlas. HPA, Human Protein Atlas; NX, consensus normalized expression.

Supplementary Figure S3 The subcellular location of ANGPTL8/betatrophin 506 based on the immunofluorescent analysis in all studied cell lines in the Cell Atlas in the HPA database. Images are available from v20.1.proteinatlas.org. HPA, Human Protein Atlas.

Supplementary Figure S4 Cancer tissue mRNA expression levels of ANGPTL8/betatrophin in the HPA database pathology atlas. HPA, Human Protein Atlas; FPKM, number Fragments Per Kilobase of exon per Million reads.

Supplementary Figure S5 Expression profile of ANGPTL8/betatrophin in the GEPIA database. GEPIA, Gene Expression Profiling Interactive Analysis; T, tumor samples; N, normal samples.

Supplementary Figure S6 Expression profile of ANGPTL8/betatrophin in the DriverDBv3 database. DriverDBv3, a database for human cancer driver gene research.

Supplementary Figure S7 Protein expression analysis of ANGPTL8/betatrophin in CPTAC samples from the UALCAN database. ANGPTL8/betatrophin proteomic expression profile in ccRCC samples grouped by (A) sample type, (B) individual cancer stage, (C) patient's race, (D) patient's gender, (E) patient's age, (F) patient's weight, (G) tumor grade. Z-values represent standard deviations from the median across samples for the given cancer type. CPTAC, Clinical Proteomic Tumor Analysis Consortium; ccRCC, clear cell renal cell carcinoma; Yrs, Years; * p<0.05, ** p<0.01, *** p<0.001 vs. normal; # p<0.05 vs. normal weight, Δ p<0.05 vs. extreme obese in FIGURE 3F; # p<0.05 vs. grade 4 in FIGURE 3G.

Supplementary Figure S8 Survival analysis of ANGPTL8/betatrophin in cancer patients from the DriverDBv3 database. Overall survival and 5-years survival of PFI survival curves of (A, B) UVM, (C, D) COAD, (E, F) UCEC were analyzed. DriverDBv3, a database for human cancer driver gene research; PFI, progression-free 535 interval; UVM, Uveal melanoma; COAD, Colon adenocarcinoma; UCEC, Uterine corpus endometrial carcinoma.

Supplementary Figure S9 Comparisons of the results on expression and survival of ANGPTL8/betatrophin through a Venn diagram. KIRC, Kidney renal clear cell carcinoma; UCEC, Uterine corpus endometrial carcinoma; LUSC, Lung squamous cell carcinoma; CHOL, Cholangiocarcinoma; LUAD, Lung adenocarcinoma; BRCA, Breast invasive carcinoma; PCPG, Pheochromocytoma and paragangliomas; SARC, Sarcoma.
