# Supplementary figures and images for: Analysis of the Expression and Prognostic Potential of a Novel Metabolic Regulator ANGPTL8/Betatrophin in Human Cancers
Source: Pathol Oncol Res. 2021 Sep 27;27:1609914. doi: 10.3389/pore.2021.1609914 (PMC8502826; doi:10.3389/pore.2021.1609914)

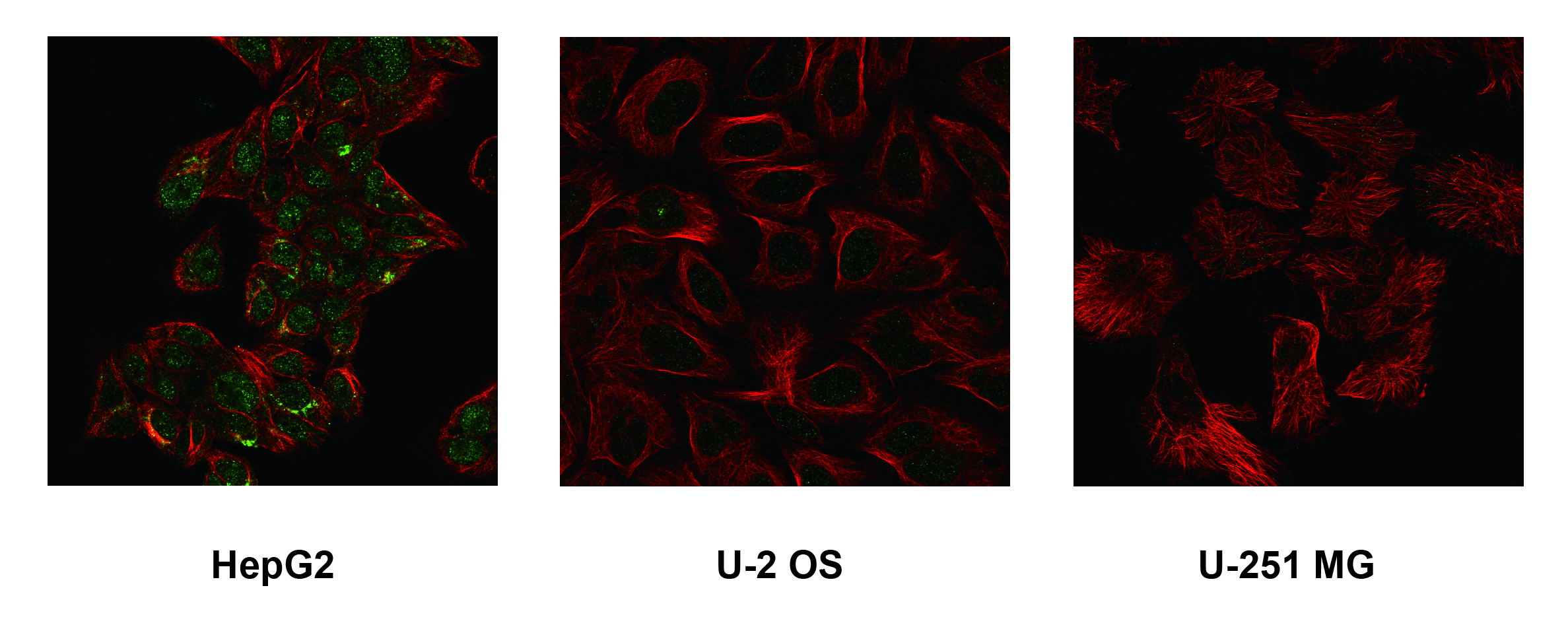

Supplement: Supplementary file 2 [file Image3.TIF]

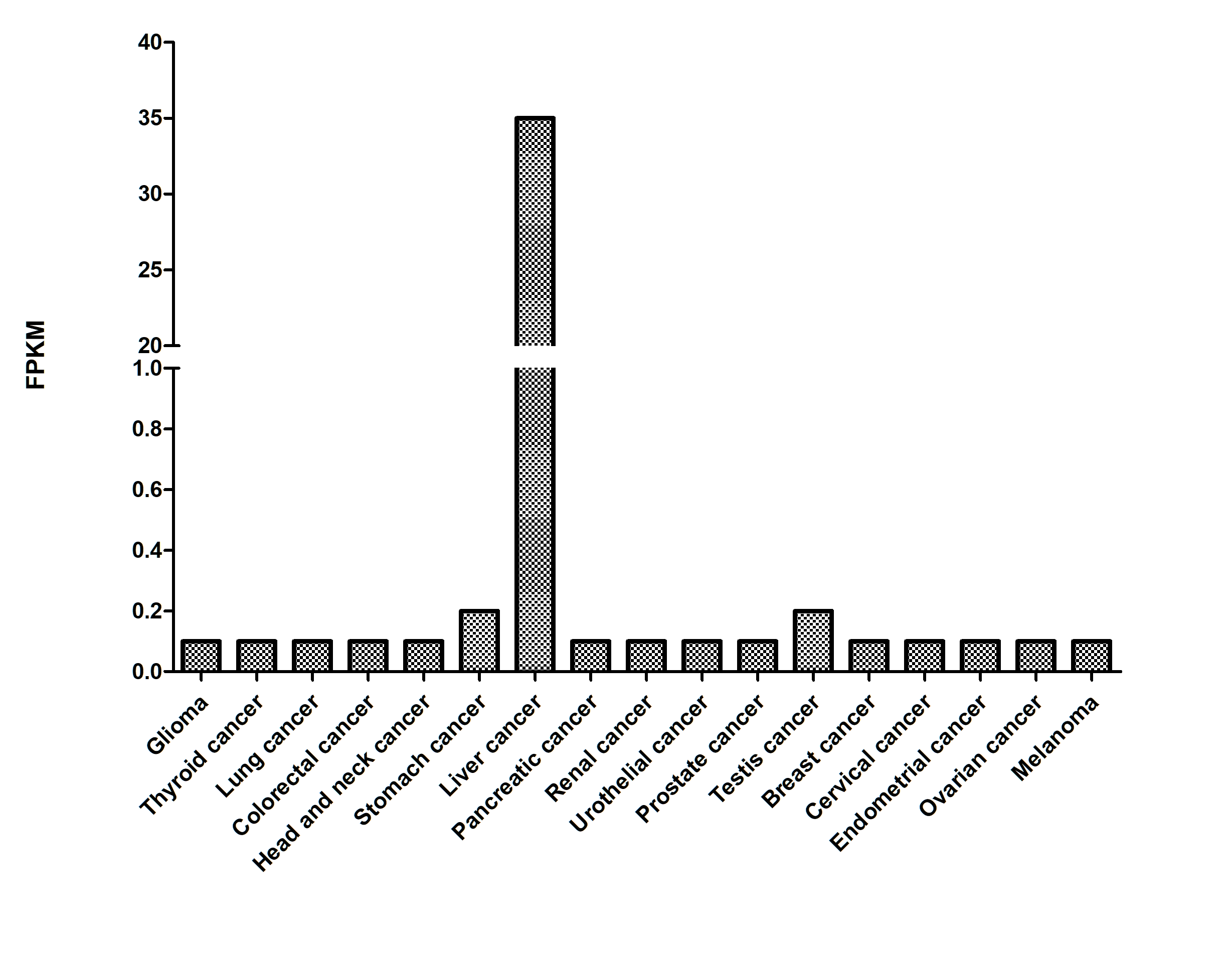

Supplement: Supplementary file 3 [file Image4.TIF]

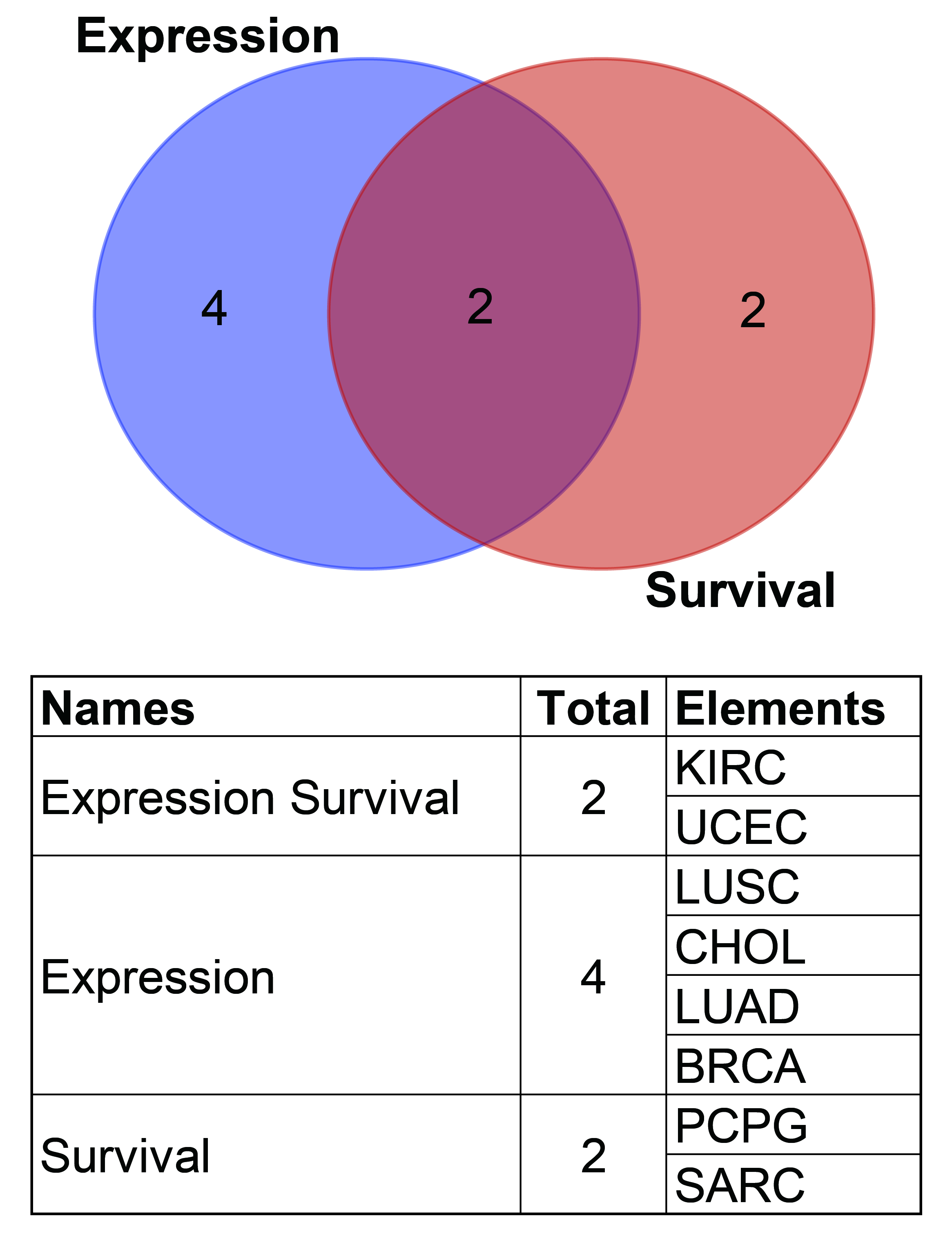

Supplement: Supplementary file 4 [file Image9.TIF]

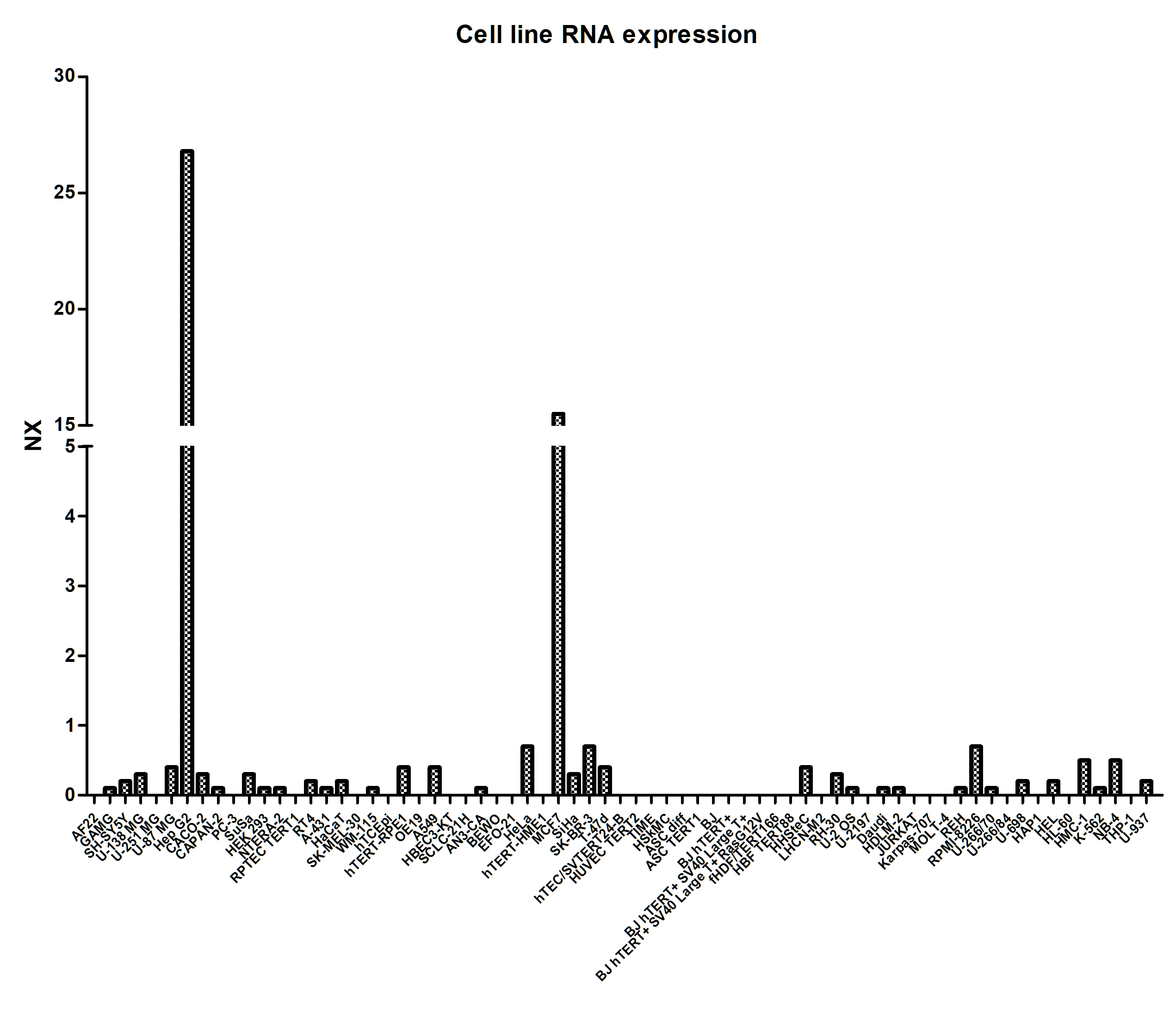

Supplement: Supplementary file 5 [file Image2.TIF]

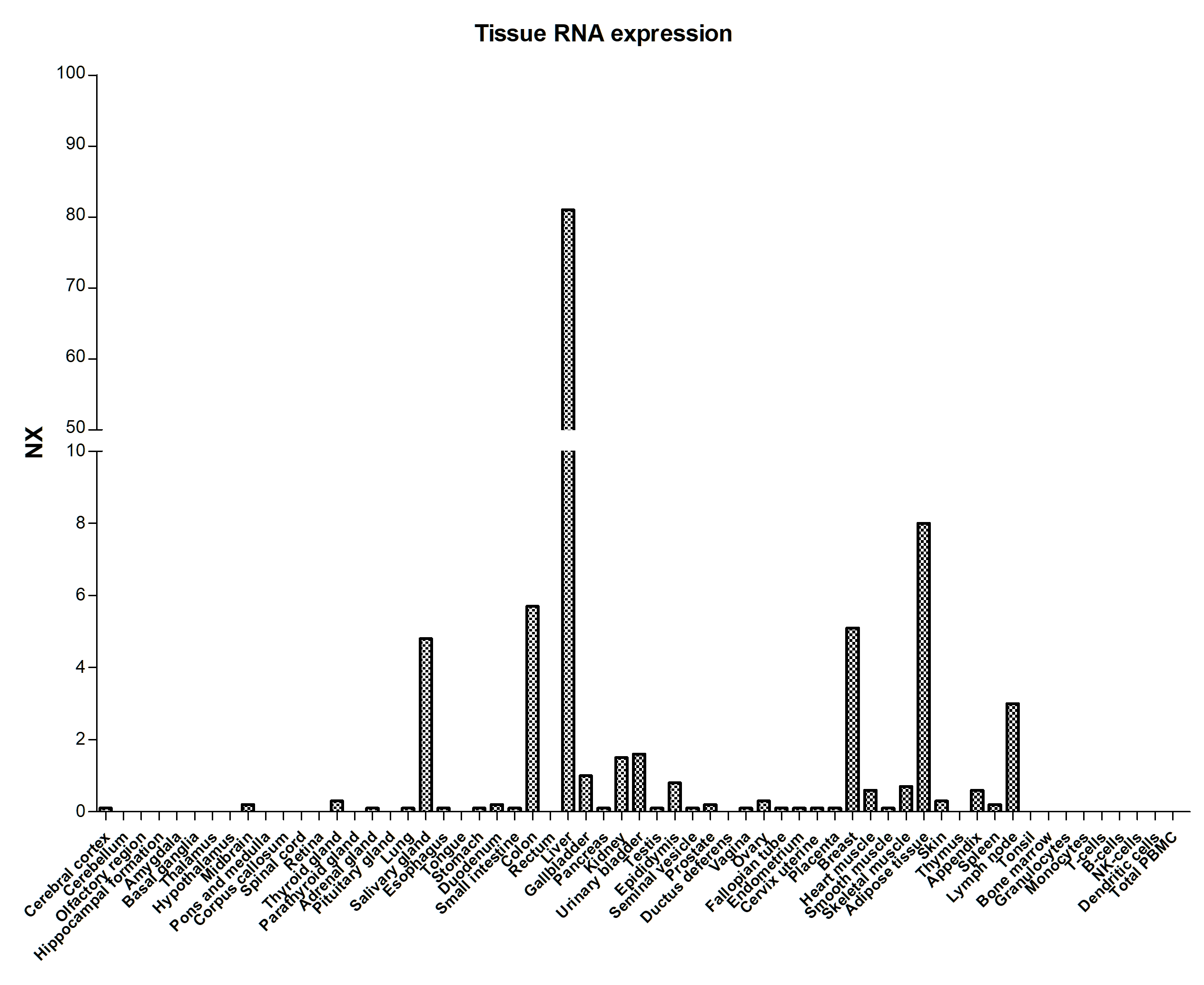

Supplement: Supplementary file 6 [file Image1.TIF]

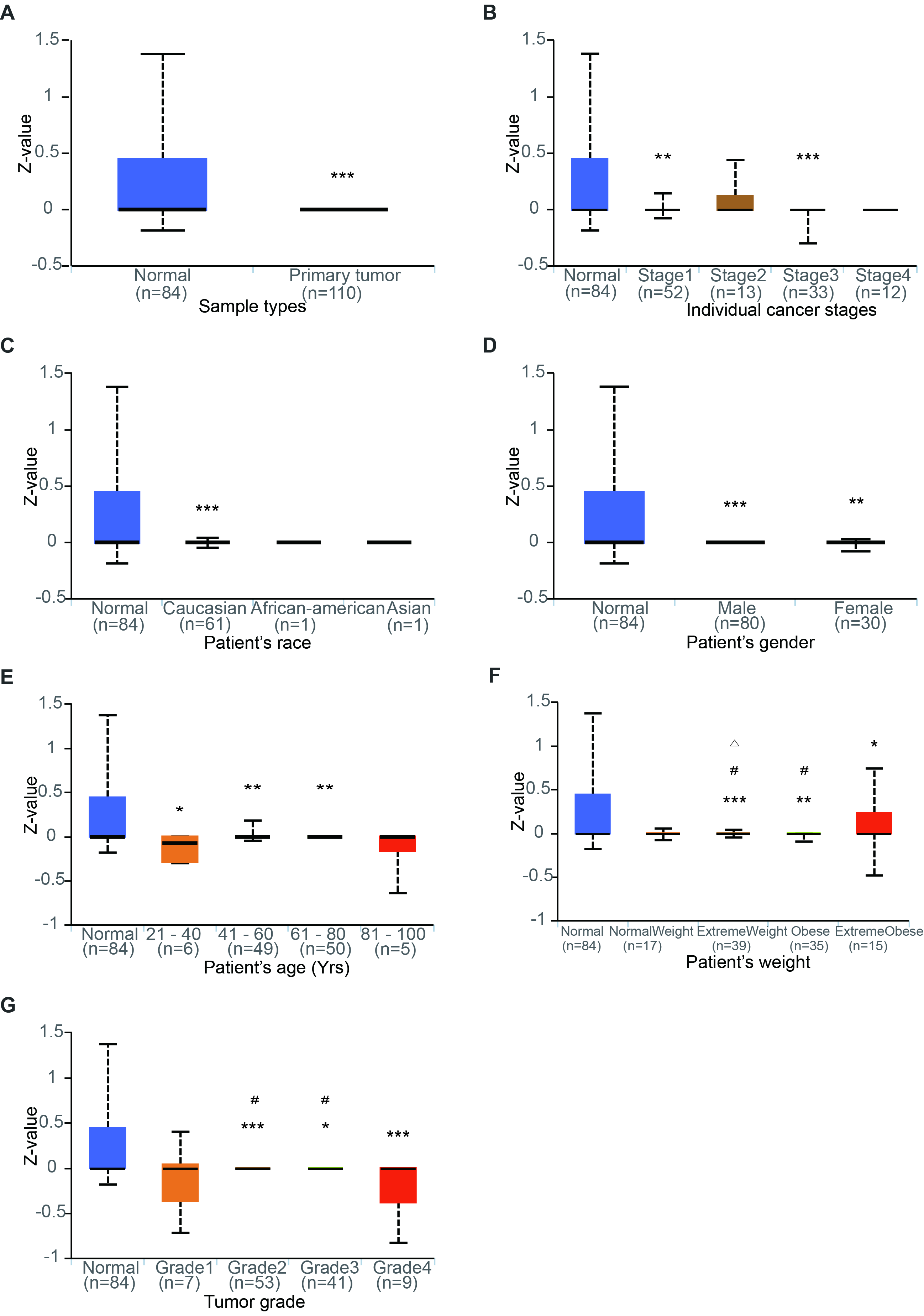

Supplement: Supplementary file 7 [file Image7.TIF]

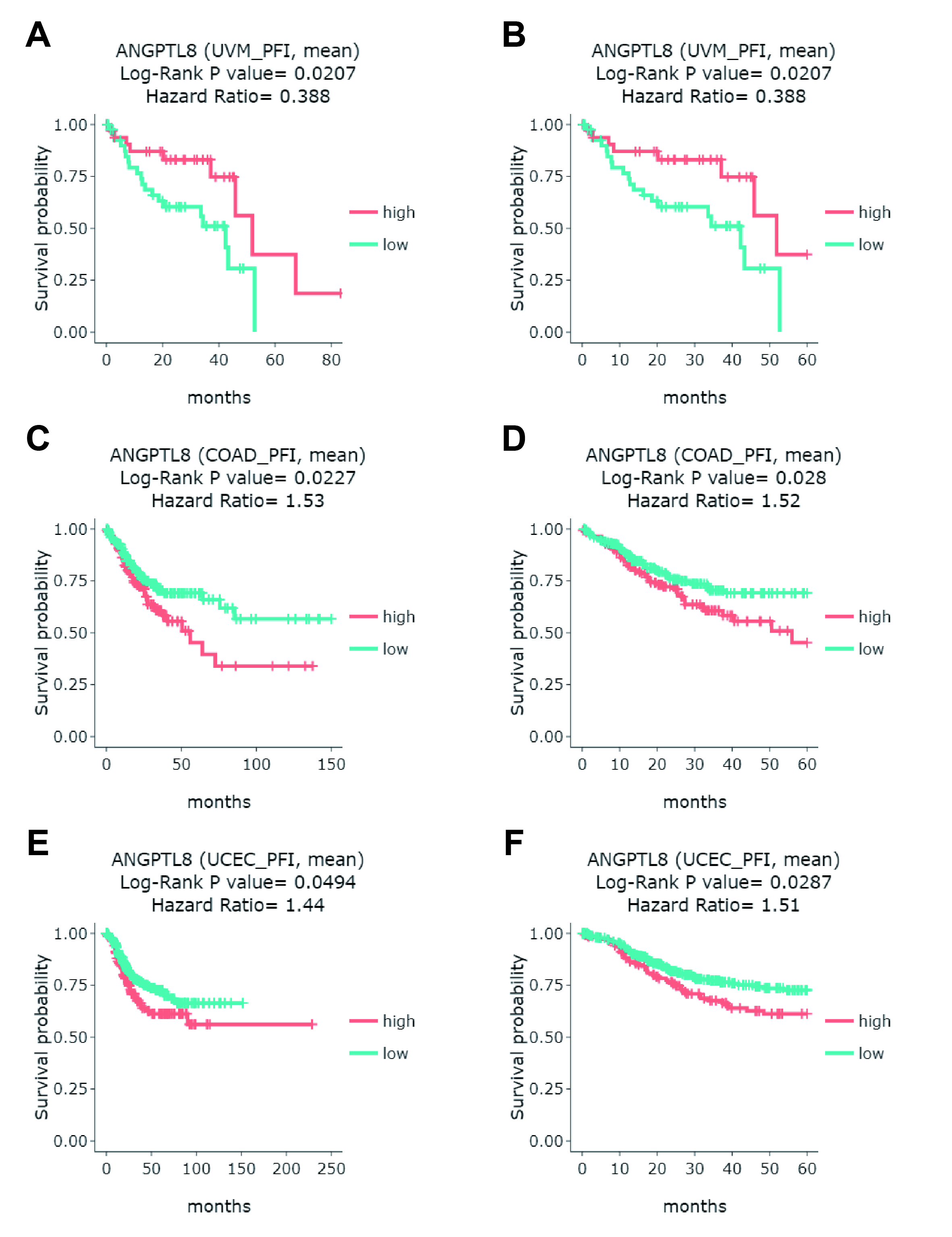

Supplement: Supplementary file 8 [file Image8.TIF]

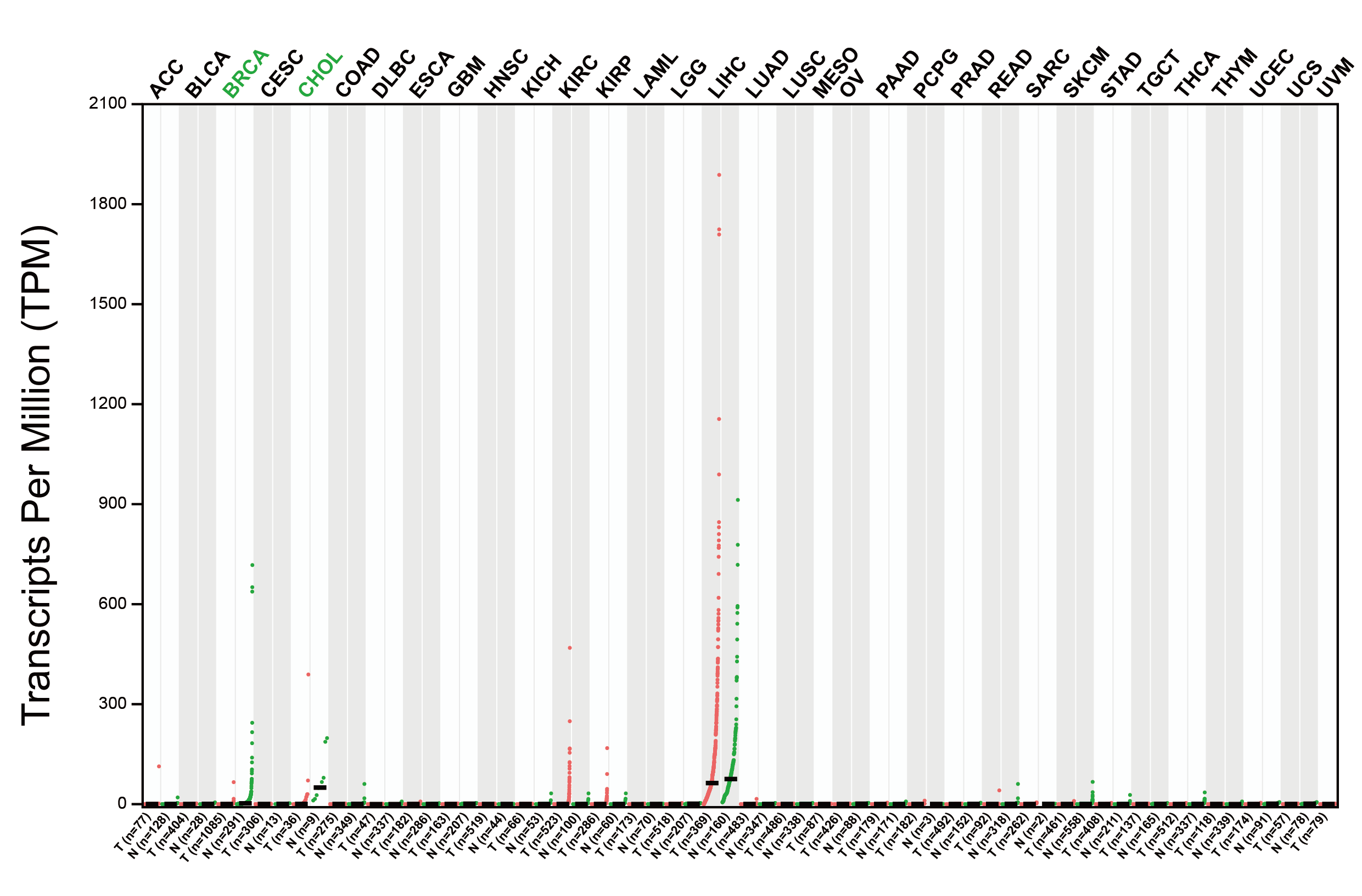

Supplement: Supplementary file 9 [file Image5.TIF]

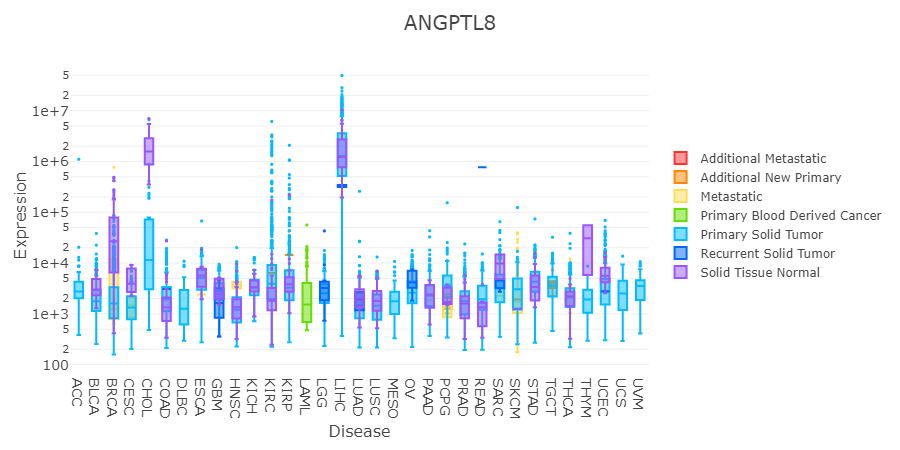

Supplement: Supplementary file 10 [file Image6.JPEG]
